# Critical Review of Exposure and Effects: Implications for Setting Regulatory Health Criteria for Ingested Copper

**DOI:** 10.1007/s00267-019-01234-y

**Published:** 2019-12-12

**Authors:** Alicia A. Taylor, Joyce S. Tsuji, Michael R. Garry, Margaret E. McArdle, William L. Goodfellow, William J. Adams, Charles A. Menzie

**Affiliations:** 1 Exponent, Inc., 475 14th Street, Suite 400, Oakland, CA 94612 USA; 2 Exponent, Inc., 15375 SE 30th Place, Suite 250, Bellevue, WA 98027 USA; 3 Exponent, Inc., One Mill and Main Place, Suite 150, Maynard, MA 01754 USA; 4 Exponent, Inc., 1800 Diagonal Road, Suite 500, Alexandria, VA 22314 USA; 5 Red Cap Consulting, 7760 North Boulder Drive, Lake Point, UT 84074 USA

**Keywords:** Copper, Regulation, Toxicity, Recommended dietary intake, Risk assessment, Essentiality

## Abstract

Decades of study indicate that copper oral exposures are typically not a human health concern. Ingesting high levels of soluble copper salts can cause acute gastrointestinal symptoms and, in uncommon cases, liver toxicity in susceptible individuals with repeated exposure. This focused toxicological review evaluated the current literature since the last comprehensive reviews (2007–2010). Our review identified limitations in the existing United States and international guidance for determining an oral reference dose (RfD) for essential metals like copper. Instead, an alternative method using categorical regression analysis to develop an optimal dose that considers deficiency, toxicity, and integrates information from human and animal studies was reviewed for interpreting an oral RfD for copper. We also considered subchronic or chronic toxicity from genetic susceptibility to copper dysregulation leading to rare occurrences of liver and other organ toxicity with elevated copper exposure. Based on this approach, an oral RfD of 0.04 mg Cu/kg/day would be protective of acute or chronic toxicity in adults and children. This RfD is also protective for possible genetic susceptibility to elevated copper exposure and allows for background dietary exposures. This dose is not intended to be protective of patients with rare genetic disorders for copper sensitivity within typical nutritional intake ranges, nor is it protective for those with excessive supplement intake. Less soluble mineral forms of copper in soil have reduced bioavailability as compared with more soluble copper in water and diet, which should be considered in using this RfD for risk assessments of copper.

## Introduction

While copper is an essential nutrient for humans, animals, and plants, it can pose risks to human health with elevated exposure. In addition to diet and supplements, a prevalent source of copper exposure in the US is through leaching of copper from plumbing into tap water. As of 2016, 78% of newly installed utility lines used copper (CDA 2016), and it is estimated that 35–40% of single-family homes newly constructed in 2019 will have copper piping (Personal communication with CDA). Copper exposure through drinking water has been regulated in the United States since 1991 (56 FR 26460-26564), when the US Environmental Protection Agency (EPA) set a maximum contaminant level goal (MCLG) and drinking water action level for copper at 1.3 mg Cu/L. EPA based the MCLG on acute gastrointestinal effects observed in nurses who consumed cocktails contaminated with copper (Wyllie 1957) (estimated minimal dose of 2.65 mg Cu/L, divided by an uncertainty factor of 2) (Donohue 1997). Although a broader review of the scientific basis for this drinking water level was conducted for EPA in 2000 (NRC 2000), which concurred with this level at the time, EPA has not since reevaluated the MCLG or formally developed an oral reference dose (RfD) to support risk assessments of environmental copper exposure. An update of the literature and an assessment of an RfD would thus be timely.

Copper has been reviewed by numerous health authorities. EPA’s Integrated Risk Information System (IRIS) evaluated copper in 1987; however, neither an oral RfD nor an inhalation reference concentration were derived (IRIS 2017), likely due to the lack of information at the time (US EPA 1988a). In 2000, EPA commissioned the National Research Council (NRC) to review the 1.3 mg Cu/L EPA drinking water action level in light of more recent experimental data on acute gastrointestinal effects in humans at that time (NRC 2000). A controlled human study reported gastrointestinal symptoms at copper levels in drinking water of ~3 mg Cu/L with no effects at 2 mg Cu/L (Pizarro et al. 1999), which was supported by unpublished data from another study sponsored by the International Copper Association. Nevertheless, NRC (2000) did not recommend increasing the MCLG because of concern for susceptible individuals with copper homeostasis disorders. The NRC reviewed both food and water ingestion studies and concluded that 1.3 mg Cu/L was protective for both gastrointestinal effects of acute exposures to elevated copper in water as well as for the rare occurrences of liver toxicosis in genetically susceptible infants or children consuming milk with elevated copper levels (NRC 2000). Subsequently, the European Chemicals Agency reviewed and endorsed a voluntary risk assessment written by the European Copper Institute (ECI) on copper and copper compounds that determined that environmentally relevant concentrations of copper were of no concern (ECI 2008).

In absence of an oral RfD, EPA and some states (e.g., New Jersey) have converted the 1.3 mg Cu/L EPA action level for drinking water to an RfD of 0.04 mg Cu/kg/day assuming 2 L/day of water consumption and a 70-kg body weight (US EPA 2017). Other state, national, and international health authorities have also developed various allowable doses for copper risk assessment, allowable dietary intake, or limits for copper in drinking water. The World Health Organization (WHO) reported that 104 countries have a regulation or guideline for copper in drinking water, with a median value of 1.5 mg/L and a range of 0.05–3 mg/L (WHO 2018).

For essential mineral elements such as copper, WHO recognized the complexity of assessing limits for copper intake and defined an acceptable range of oral intake as the trough in a U-shaped curve that balances deficiency of low exposure with toxicity from excess exposure (WHO 2002). Although an oral RfD is not intended to provide essential intakes for good health, an RfD in the range of deficiency is lower than necessary for protection against toxicity from excess. Derivation of an oral RfD for copper thus needs to consider toxicity data in animals, human experimental studies, and epidemiological studies, along with the mechanistic effects of copper as an essential element in biological functions, tolerance over a wide range of doses from internal regulation of copper levels, and toxicity that results from excess internal copper, especially for some uncommon genetically susceptible groups.

Though increasing copper exposure in the US population is not a concern, a better understanding of the levels of essentiality and toxicity is needed to support the scientific basis of health-protective regulations for this element. The objectives of this review are to summarize the current knowledge on copper toxicity, assess dose-response endpoints, and provide useful information for health professionals, regulatory agencies, and policy makers for assessing the health risks of copper and determining whether current research warrants reevaluation of copper criteria for risk assessment and risk management of environmental exposures.

## Methods

### Literature Search, Review, and Evaluation Methods

The most recent authoritative reviews on copper conducted by WHO (2004) and ECI (2008) were initially evaluated with a critical review of the key studies identified by these reports. A literature review was also conducted to identify copper toxicity studies published from 2007 to 2019 using the National Library of Medicine database, PubMed, the Developmental and Reproductive Toxicology Database,^1^Hazardous Substances Data Bank, International Toxicity Estimates for Risk, and Google Scholar. Broad search terms relating to various forms of copper and, health endpoints, and relevant dose/exposure information were used. Google scholar was also used with broad search terms relating to copper and health, to find additional publications. Over 260 publications were identified using all of the search engines and were reviewed for relevance.

### Dose-Response Assessment Methods

Key studies selected in the above screening process were reviewed for data quality and potential relevance for developing a robust oral RfD for copper. Papers were selected on oral toxicity endpoints traditionally used for the analysis in EPA’s IRIS process (IRIS 2017), including in two-generation laboratory animal studies, double-blind studies, and those with appropriate controls. Previous studies have evaluated best methods for determining an optimal copper intake (Chambers et al. 2010) and identify study designs needed to best model copper toxicity (Krewski et al. 2010a, 2010b). Specifically, an alternative nonlinear relationship used for essential nutrient toxicity that simultaneously models both deficiency and excess toxicity dose-response curves, called categorical regression, was reviewed as a potential method for defining optimal copper intake levels (Chambers et al. 2010; Krewski et al. 2010a, 2010b; Milton et al. 2017a). More details on the categorical regression methodology are presented in limitations of deriving an EPA RfD section. Consistency with the broader literature on the mode of action of copper essentiality and toxicity and derivations of limits for copper intake or drinking water concentrations were also considered in assessing an appropriate oral RfD.

## Chemical Substances, Properties, and Uses

A description of copper substances, properties, and uses is provided in section 1.0 of the Supplementary Information and in Supplementary Table S1. When possible, the form of copper is provided that was used in the publications summarized herein.

## Essentiality and Homeostasis

Copper is an essential trace element necessary for all biological organisms and its levels are controlled in the body. The dysregulation of copper through impaired absorption or excretion results in deficiency or toxicity, as illustrated by two rare genetically based diseases.

### Role of Copper Essentiality and Normal Regulation

In animals and plants, copper is found in many enzymes and has two main functions (ATSDR 2004; ECI 2008): (1) in fundamental reduction–oxidation reactions involving Cu(II) and Cu(I), such as in superoxide dismutase, hemocyanin, and cytochrome c oxidase, and (2) as a component in structural and functional proteins because of its electron transfer capabilities, with examples including neurotransmitter function, iron metabolism, and connective tissue biosynthesis (IPCS 1998). Copper-containing enzymes in humans, including but not limited to metallothionein, tyrosinase, hemocyanin, ceruloplasmin, and amine oxidase, are important in multiple systems, such as the respiratory, immune, and nervous systems. Cytochrome c oxidase, for example, is essential for brain function and energy generation in the brain (Llanos and Mercer 2002).

As summarized by Llanos and Mercer (2002), most copper homeostasis mechanisms are controlled by Cu-ATPases, which allow cells to efflux excess copper as cupro-proteins. For example, the Cu-ATPase ATP7A is a transporter necessary for copper uptake across the small intestine and across the blood–brain barrier. Specifically, ATP7A functions as a cellular copper efflux protein, whereas ATP7B is responsible for copper biliary excretion or transport from the liver to ceruloplasmin. Other proteins are involved along with a nonspecific metal–ion transporter, DCT1, which is thought to play a minor role in dietary copper absorption.

Copper essentiality is demonstrated by its role in many physiological processes, such as fetal and infant development and growth, brain development, and function, bone strength, iron, glucose, and cholesterol metabolism, pigment formation, and immune function (ECI 2008). Copper requirements are met through ingestion of copper in water and foods. Average concentrations of copper found in the US tap water are between 0.02 and 0.075 mg/L (ATSDR 2004). Gaetke et al. (2014) estimated that drinking water contributes ~6–13% of the average daily copper intake in the US Food sources of dietary copper include organ meats, nuts, seafood, seeds, beans, and whole grains (ATSDR 2004; WHO 2004). Median intake of copper from food sources is 0.93–1.3 mg/day for adults in the United States (ATSDR 2004). The US Institute of Medicine (IOM) also provides dietary intake values: the mean dietary intake from food alone is estimated at 1.0–1.7 mg Cu/day, whereas the mean dietary plus supplemental copper intake is 1.3–2.2 mg Cu/day (IOM 2001). Up to 15% of US adults consumed dietary supplements containing an average of 1.3–2.2 mg Cu/day as of 1986 (IOM 2001). Using average intake values of copper from water, food, and supplements, the average adult may consume an approximate total of 1.85 mg Cu/day, or equivalent to 0.026 mg Cu/kg/day.^2^ Dietary reference values for copper have been recommended (IOM 2004), although the exact amounts of copper associated with adequate nutrition, deficiency, and toxicity have yet to be defined (Bost et al. 2016).

The essentiality of metals complicates the assessment of a RfD for health protection because of toxicity versus recommended dietary allowances for adequate nutritional requirements (US EPA 2007). Nutritional requirements vary across the population. Differences in homeostatic copper regulation also affect individual nutritional requirements and toxicity levels. Studies in monkeys and rats suggest that neonates are more sensitive to toxicity from copper exposure because of less competent regulatory capabilities that further develop with age (Fuentealba et al. 2000; Araya et al. 2005; Bauerly et al. 2005), although the tendency to accumulate more copper is offset by increased requirements in this early rapid growth period. Rare homeostatic disorders like Menke’s and Wilson’s diseases are examples of hereditary diseases that alter essentiality and deficiency/toxicity levels in humans.

### Menke’s and Wilson’s Diseases

Failure to maintain copper homeostasis within the body leads to copper deficiency or excess, both of which result in toxicity. Two hereditary diseases in humans, Menke’s disease, which is a deficiency disorder, and Wilson’s disease, an excess disorder, demonstrate the consequence of copper dysregulation in humans (Danks 1995; ECI 2008). Both diseases result from genetic defects in copper transport genes for Cu-ATPases (Llanos and Mercer 2002).

Menke’s disease is an X-linked copper deficiency syndrome that occurs in 1 out of 250,000 live births (Llanos and Mercer 2002; Tønnesen et al. 1991). Impaired copper delivery out of intestinal cells by the regulatory protein Cu-ATP7A in patients causes systemic copper deficiency characterized by hypopigmented hair and connective tissue abnormalities leading to aneurisms, loose skin, and fragile bones (Llanos and Mercer 2002). Defective transport by ATP7A also results in impaired copper distribution throughout the body, including the lack of normal transport across the blood–brain barrier resulting in neurologic deficiencies (Llanos and Mercer 2002). Inadequate copper transport also results in excess copper in the kidneys, consistent with deficient copper efflux from cells as the overall defect of this disease. Menke’s disease presents in patients at 2–3 months and is usually fatal by 3 years (de Bie et al. 2007).

Wilson’s disease is an autosomal recessive disorder of copper transport in 1 out of 50,000–100,000 live births (Llanos and Mercer 2002; Danks 1995). A nonfunctional ATPase, ATP7B, results in high copper concentrations in the liver due to an inability to excrete biliary copper, causing oxidative damage, hepatocyte death, and liver failure (Llanos and Mercer 2002). Damaged hepatocytes release copper into circulation, which can accumulate in the central nervous system leading to neurotoxicity (ataxia, dementia, anxiety, and disorientation; Llanos and Mercer 2002). No overt symptoms are apparent during the gradual accumulation of copper within the body, which is then followed by a sudden onset of toxicity as hepatocytes can no longer store or complex the excess copper which accumulates in the tissues (Llanos and Mercer 2002). Onset of Wilson’s disease can occur between the ages of 8–9 and early 50s, though most commonly patients are diagnosed with liver dysfunction as a teenager (DiDonato and Sarkar 1997; Goode 1991). There is a broad spectrum of clinical symptoms associated with Wilson’s disease, and therefore making diagnosis a challenge (Beinhardt et al. 2014). Wilson’s disease patients often need liver transplants due to liver cirrhosis (Beinhardt et al. 2014). Even patients receiving adequate treatment will have a reduced life expectancy compared with healthy individuals (Beinhardt et al. 2014). Copper has been linked to neurological effects in Wilson’s disease patients, although Gromadzka et al. (2010) did not find clear evidence of increased neurological effects in the 68 heterozygotes for Wilson’s disease tested when compared with 31 control subjects.

These two diseases illustrate the consequences of the adverse effects of deficient or excess copper in the body in genetically susceptible populations; however, these conditions are not informative of the oral doses for prevention of toxicity or deficiency. Those with Wilson’s disease would have toxic accumulation of copper with even the low levels of exposure necessary for good nutrition in the general population, and are a rare subpopulation that would be under medical care. The heterozygous carriers of the Wilson’s disease gene are more common in the population and have been postulated to potentially have genetic susceptibility to expressing some Wilson’s disease symptoms with elevated copper exposures (Brewer 2000). Genetic susceptibility has also been indicated in idiopathic liver toxicosis cases in India (NRC 2000; Nayak and Chitale 2013; Uauy et al. 2008; Sriramachari and Nayak 2008; O’Neill and Tanner 1989) and the Tyrolean region of Austria (NRC 2000; Müller et al. 1996; Uauy et al. 2008), as discussed in the section relating to liver toxicosis. Intake of copper by individuals with Wilson’s (homozygous) and Menke’s diseases is likely managed by their medical professionals because of the overt symptoms of these diseases, even at low copper exposure in the case of Wilson’s disease. Thus, those with these diseases have not been considered for setting regulatory levels (NRC 2000).

## Toxicokinetics

### Absorption and Elimination

In mammals, copper absorption occurs in the upper gastrointestinal tract through active and passive transport mechanisms (WHO 2004). Copper absorption primarily occurs in the small intestine and is susceptible to competitive inhibition from transition metals such as iron or zinc (Llanos and Mercer 2002; WHO 2004). In fact, zinc is used to treat Wilson’s disease via copper absorption inhibition (IOM 2001). Numerous studies have investigated copper absorption in animals and humans following oral exposures.

Absorption of copper through active transport primarily involves the copper transporter 1 (Ctr1) that is specific for transporting the monovalent (Cu(I)) form of copper into cells (including gastrointestinal cells). Copper in food can be either Cu(I) or divalent (Cu(II)) forms as Cu(I) acetate, Cu(II) acetate, Cu(I)-glutathione, Cu(I)-cysteine, and Cu(II)-histidine; whereas, copper in water is as Cu(II) (Ceko et al. 2014). Ctr1 is critical for development since its deletion is associated with lethality in animals (Lee et al. 2001). Active mechanisms for copper absorption from small intestine, particularly in the duodenum, involve transport through Ctr1 into the enterocytes of the duodenum. Ingested Cu(II) is readily reduced to Cu(I) by metalloreductase activity at the apical membrane of the gastrointestinal enterocytes (Ohgami et al. 2006; Ellingsen et al. 2015). Changes in absorption rate depending on the cellular copper status are likely related to the activity/efficiency of this enzyme and transporters like Ctr1. Higher copper exposure results in internalization of Ctr1 within the enterocytes, thereby reducing transport (Lonnerdal 2008; Van den Berghe and Klomp 2009). Ctr1 on the basement membrane side of the enterocyte can also transport copper into the enterocyte from the blood. Normal shedding of enterocytes (4-day average lifespan) into the gastrointestinal tract results in another source of elimination for the regulation of copper. Copper delivered to portal circulation is bound to histidine or serum proteins such as α2-macroglobulin and albumin (Van den Berghe and Klomp 2009).

Absorption in the gastrointestinal tract may also occur by diffusion through tight junctions (paracellular), although passive absorption in this manner would be less efficient than facilitated transport. Unlike calcium and magnesium absorption, little evidence supports passive paracellular uptake of copper as an important pathway for absorption under normal conditions, consistent with the lack of intestinal absorption of copper in Menke’s disease and rats lacking Ctr1 (Van den Berghe and Klomp 2009). On the other hand, Van den Berghe and Klomp (2009) acknowledge that paracellular transport cannot be ruled out under conditions of “superphysiological” copper intake, and that some evidence indicates preadolescent rats rely on paracellular uptake of copper, although they also show regulation of blood copper levels through accumulation in intestinal cells. Clearly, additional research is needed on the conditions in which paracellular transport of copper (including Cu(II)) might be important.

The amount of copper absorbed by Ctr1 or by passive absorption may thus depend on the dose, whether one is copper deficient or sufficient, and gastrointestinal transit time. For example, administration of radiolabeled Cu(II) (0.5 mCi ^64^copper acetate; dose in µg not given but appears to be 61.7 µg^3^ or 0.26 mg/L if administered in 8 oz of fluid) in cow’s milk to fasting adults resulted in peak uptake into blood of 6% of the administered dose within 1–2 h of ingestion (Hill et al. 1986). The rapid appearance of copper in blood is likely related to the administration of copper in a soluble form under fasting conditions. Some of the absorption may have occur through reduction of Cu(II) to Cu(I) and the Ctr1 transporter in the duodenum, although rapid gastrointestinal transit time under these conditions would have reduced the time for such transport. Although total copper absorption was not reported, a low peak absorption within 1–2 h suggests an overall low absorption percentage and little evidence of high exposure from passive absorption in the lower gastrointestinal tract.

Van den Berg et al. (1994) showed using radiolabeld ^64^Cu, that oral ingestion of 5 µg of Cu administered in feed by fasting Wistar rats compared with 5 µg delivered by intraperitoneal administration of a solution resulted in total absorption values of 52–56%. When the same conditions were tested with a copper deficient diet (1 mg/kg of Cu in feed), 58–76% absorption occurred. In another study, where male Long Evans rats were orally administered adequate copper or copper deficient diets, absorption rates were shown as 32–39% for both diets, though absorption values may not reflect complete excretion (Johnson 1988). Other absorption research indicates that rats adjust to deficient or low copper doses by increasing copper absorption efficiency and reducing biliary excretion; rats on increased copper diets show a decreased absorption percentage and increased fecal excretion (ECI 2008).

In human studies, Turnlund et al. (1989) administered 12 young men with three different copper dietary doses. Mean absorption values were ~55% for 0.9 mg Cu/day, 36% for 1.99 mg Cu/day, and 12% for 7.78 mg Cu/day. Similar to the rat studies mentioned above, the results indicate that decreased copper absorption occurs with increasing dietary copper. Over the course of the experiments, the overall internal copper balance was maintained at a relatively constant rate. This study shows that internal copper levels are first regulated through absorption, with a second regulatory action through biliary excretion and fecal loss. In a similar study, Turnlund et al. (2005) performed a long-term copper dietary experiment in nine men (ages 26–49). Absorption ranged between 29 and 40% and was dependent on the dose of copper given, with higher absorption at the lower dose (0.65–2.2 mg Cu/day). Studies have also been conducted on the effects of other dietary constituents on absorption, such as zinc, ascorbic acid, and fiber, in which no clear associations were found (ECI 2008), though some studies indicate that zinc and iron are antagonistic to copper absorption (ECI 2008).

Numerous studies indicate that dietary trace mineral exposure is affected by the mineral content and its bioavailability in different food matrices. Vegetarian diets are reported to contain more trace minerals, such as copper and manganese, compared with non-vegetarian diets (Hunt 2003). However, research indicating no differences in copper plasma levels between vegetarians and non-vegetarians or even higher Cu exposures from vegetarian diets, likely reflect differences in bioavailability as well as copper concentration among food types (Krajčovičová-Kudláčková et al. 1995; Hunt 2003; Van den Berghe and Klomp 2009). Earlier work indicated high amounts of fiber and phytate in plant-based foods reduces copper bioavailability compared with meat-based diets (Srikumar et al. 1992b). Based on this finding and ~25% reduction in copper bioavailability in two Wilson’s disease patients on a vegetarian diet, vegetarian diets have been proposed as a part of treatment for Wilson’s disease (Brewer et al. 1993). Nevertheless, in studies in rats, cooked food with high protein content had lower copper absorption when compared with vegetable and other meat sources (Johnson and Lee 1988; Wapnir 1998), whereas a high-meat diet resulted in higher excretion of copper in the feces compared with the low-meat diet, likely related to increased biliary excretion (Hunt et al. 1995). Hunt et al. (1995) indicated that, despite changes in copper availability based on diets, homeostasis maintains a relatively constant copper balance. Bost et al. (2016) reported that a range of dietary copper intake (0.57–68 mg/day) did not affect copper concentrations in plasma.

Other health issues, such as hypertension, also impact copper plasma levels (Srikumar et al. 1992a). This body of research (Hunt et al. 1995; Hunt 2003; Johnson and Lee 1988; Krajčovičová-Kudláčková et al. 1995; Srikumar et al. 1992b; Wapnir 1998) indicates that diet, bioavailability, and copper dysregulation may have various impacts and potential interactions affecting copper uptake and essentiality. ECI (2008) determined that, based on limited information on bioavailability and the absorption rate of other copper compounds, copper sulfate (CuSO_4_) (which is one of the more soluble forms) can be used as representative for copper compounds when conducting risk assessments. More information on bioavailability and water solubility, such as for multiple forms of copper, can be found in section 2 of the Supplementary Information.

Fecal elimination after biliary secretion of copper complexed to organic ligands is documented as the primary route of copper excretion in laboratory animals (ECI 2008; Johnson 1988; Van den Berg et al. 1994). An estimated 80–90% of biliary copper is excreted in the feces in humans (Winge and Mehra 1990). Winge and Mehra (1990) also demonstrate that fecal elimination is a key to maintaining homeostatic copper levels in the liver and is a more important mechanism for copper regulation than absorption. No studies have measured elimination through urine, saliva, sweat, or hair according to ECI (2008) and our review of subsequent literature. It appears that little additional information quantifying absorption and elimination percentages has been published in the past decade. In addition, no publications were found that discuss what proportion of copper forms, (Cu(I) and Cu(II), is absorbed by the gastrointestinal tract through active or passive transport pathways.

### Distribution and Metabolism

Laboratory animal studies support a two-stage model of copper transport. As copper enters the bloodstream after absorption in the gastrointestinal tract, ionic copper complexes with plasma proteins such as albumin, ceruloplasmin, and transcuprein and is transported through portal circulation to the liver (NRC 2000). Ceruloplasmin carries 60–95% of copper found in the blood serum (IOM 2001). While normal values for ceruloplasmin and blood serum have been identified, these measures are subject to rapid changes based on diet and have not served as reliable biomonitoring indicators of exposure. The normal range of copper found in blood serum is 0.635–1.59 mg Cu/L (IOM 2001). Albumin is the most abundant blood protein with a high affinity and capacity for copper binding (600 mg/L of blood plasma), which together with other metal binding proteins, results in very little “free” ionic copper in blood (Linder 2016). Uptake of albumin-bound Cu(II) across cellular barriers also occurs via cell surface reductases and Ctr1 and other unidentified transporters (Lee et al. 2002; Linder 2016). The liver also complexes any free copper ions with proteins that are then secreted into the bloodstream, stored in the liver, or excreted in bile to the gastrointestinal tract, thereby maintaining homeostasis (ECI 2008). Within a cell, copper is stored complexed to a metal binding protein, metallothionein. In addition, proteins found in the cell cytoplasm, called metallochaperones, bind copper and prevent the release and accumulation of the more toxic free copper ions within a cell, while providing necessary copper delivery to intracellular locations (ECI 2008). As noted above for Menke’s and Wilson’s Dieases, the regulatory proteins ATP7A and ATP7B, respectively, are instrumental in transporting copper out of cells (e.g., from intestinal cells to portal circulation and across the blood–brain barrier for ATP7A; from the liver into bile for ATP7B).

Numerous distribution and metabolism studies have been conducted in mammals. In Sprague–Dawley and Fisher female rats, copper is first found bound to albumin with peak liver concentrations at 6 and 24 h, representing 2–40% of the dose. In parallel, an increase of copper with ceruloplasmin was measured. After 24 h, no copper was measured in albumin. Throughout the study, copper-ceruloplasmin represented 50% of the dose, and >90% of the dose was found in plasma proteins, including ceruloplasmin. Copper was also detected in other organs besides the liver and kidney, such as the skeletal muscle (18%), brain (2–3%), and heart (8%) (Weiss and Linder 1985).

The distribution and metabolism of copper in humans is similar to that found in animals. The liver and brain are the organs with the highest concentrations of copper with muscle overall containing the largest amount of copper on a total mass basis (ECI 2008).

## Noncancer Toxicity

### Acute Toxicity

Copper toxicity typically results from the production of reactive oxygen species during redox reactions involving excess free or ionic copper forms, and from sufficient accumulation to overwhelm protein-binding capacity (Gaetke et al. 2014). In humans, acute effects of copper ingestion include gastrointestinal symptoms such as nausea or abdominal pain. Olivares et al. (2001) gave men and women (*n* = 61, total) a single dose of 0–12 mg Cu/L as CuSO_4_ in deionized drinking water using a randomized design, followed by collection of self-reported perceived symptoms at 15 and 60 min and 24 h. Nausea was most commonly reported immediately following the dose, and 12 mg Cu/L had the highest percentage of nausea reported (21%). The lowest-observed-adverse-effects level (LOAEL) was 4 mg Cu/L, and the no-observed-adverse-effects level (NOAEL) was 2 mg Cu/L. Nausea was reported in 76% of the volunteers.

Studies by Araya et al. (2001, 2003a) are the most comprehensive investigations of the levels of copper in water that cause acute gastrointestinal effects. These studies were used by ECI (2008) to derive the LOAEL and NOAEL used for copper in drinking water. Araya et al. (2001) used a similar study design as reported in Olivares et al. (2001). Doses of copper were given once a week for 5 weeks at 0–8 mg Cu/L to a total of 179 volunteers (men and women). Up to 18% of volunteers reported nausea at the 8 mg Cu/L dose. In Araya et al. (2003a), a total of 249 women in Chile, the United States, Northern Ireland, and China were administered a single, oral bolus dose in bottled water at 0, 2, 4, 6, or 8 mg Cu/L. The study was double-blind with each person as their own control. Consistent with the other two studies, nausea was the most common and earliest symptom in Araya et al. (2003a) and occurred within 15 min of ingestion. Reports of nausea increased with higher doses, and nausea was the most frequently reported symptom. The LOAEL for nausea was 6 mg Cu/L and the NOAEL was 4 mg Cu/L. Other symptoms were reported less frequently and included abdominal pain, vomiting, and/or diarrhea. Gastrointestinal effects occurred immediately and stopped once the exposure ceased (Araya et al. 2003a). These controlled human experimental trials conducted with multiple international populations allow for a reliable identification of a copper concentration in drinking water that does not cause irritation to the gastrointestinal tract leading to symptoms.

### Irritation and Sensitization

Irritation and sensitization associated with skin contact are less relevant for assessing risk from copper via the oral exposure pathway. Stomach irritation from water ingestion is addressed in the sections relating to acute toxicity and repeated-dose toxicity. Sensitization, whether oral or dermal, does not appear to be relevant for copper (ECI 2008). An additional summary of data on irritation and sensitization is provided in section 3.0 of the Supplementary Information.

### Repeated-Dose Toxicity

#### Human experimental studies

Several repeated-dose studies of copper exposure in drinking water or in other soluble forms have been conducted in humans (summarized in Table 1). Additional details on the human water ingestion studies are presented in Supplementary Table S2. One of the most robust studies is a cohort study in Chile (Olivares et al. 1998) in which 128 formula- or breast-fed infants, 3–12 months old, were assigned to a copper-supplemented (2 mg Cu/L in drinking water using CuSO_4_) or control group (<0.1 mg Cu/L). Drinking water for each group was also used in making formula and in food preparation. Formula-fed infants were weaned at 3 months, and breast-fed infants started solid food at 6 months, with interim formula provided when necessary. Drinking water for the mothers contained 2 mg Cu/L and the control contained 0.1 mg Cu/L. The water concentration of 2 mg Cu/L was selected for this study based on the WHO guideline at the time. Mothers of the breast-fed infants also consumed the same water concentrations as the infant. Water consumption was recorded, and blood samples at 6, 9, and 12 months were analyzed for biochemical parameters (serum copper level, ceruloplasmin level, erythrocyte enzyme activity, and metallothionein), liver function (enzymatic activity, serum bilirubin), gastrointestinal symptoms, and respiratory disorder. Infants in the 2 mg Cu/L group had elevated ceruloplasmin activity at 9 months. No effects were observed for growth or morbidity. A NOAEL of 2 mg Cu/L in drinking water was determined with endpoints of biochemical indicators for liver toxicity and gastrointestinal symptoms; however, participant dropout was higher in the 2 mg Cu/L group, which may have resulted in under-reporting of symptoms in this study.

**Table 1:** Summary of human ingestion studies

Group	Cu form	Treatment	Water concentration or dose (mg/L or mg/day)	Results summary	Cu NOAEL or LOAEL	Reference
Women (*n* = 60)	CuSO_4_	0, 1, 3, and 5 mg/L in tap water for 2 weeks followed by 1 week of tap water without copper	0, 1, 3, and 5	Acute (GI) symptoms increased at >3 mg/L, no significant differences in effects between different copper ratios	NOAEL: 2 mg/L	Pizarro et al. (1999)
Adult women (*n* = 45)	Copper sulfate: copper (II) oxide ratios	5 mg/L in tap water for 1 week followed by 1 week without copper in tap water, alternating for a total of 9 weeks, double-blind study	Ratios of soluble (copper sulfate) to insoluble (copper (II) oxide): 0:5, 1:4, 2:3, 3:2, and 5:0	Liver enzymatic function not affected, increase in GI symptoms, 6/12 diarrhea during first week, both copper compounds caused 54 or 18% nausea incidence in water or orange juice, respectively	ND	Pizarro et al. (2001)
Women (*n* = 269)	CuSO_4_	Single dose in bottled water	0, 0.4, 0.8, 1.2, and 1.6 mg Cu in 200 mL bottled spring water (0, 2, 4, 6 and 8)	Nausea first/most common symptom, within 15 min of ingestion	LOAEL (nausea): 6 mg/L; NOAEL 4 mg/L	Araya et al. (2003a)
Men and women (*n* = 179)	CuSO_4_	Weekly dose for 5 weeks	0, 2, 4, 6, and 8	Nausea first/most common symptom, within 15 min of ingestion	LOAEL: 6 mg/L; NOAEL 4 mg/L, both for GI effects and nausea	Araya et al. (2001)
Men and women (*n* = 1365)	CuSO_4_	Daily dose for 2 months, water prepared for drinking and used in food preparation	<0.01, 2, 4, and 6	Gastrointestinal effects increased at 6 mg/L, no effects for other endpoints	LOAEL (nausea) 6 mg/L	Araya et al. (2003b)
Men and women (*n* = 61)	CuSO_4_	Weekly dose administered in water or orange-flavored juice for up to 12 exposures	0, 2, 4, 6, 8, 10, and 12	Nausea and vomiting reported	In water NOAEL (nausea): 2 mg/L; NOAEL (vomiting): 4 mg/L; in orange-flavored drink NOAEL (nausea): 8 mg/L, LOAEL (nausea): 4 mg/L	Olivares et al. (2001)
Men and women (*n* = 7)	Copper gluconate	1 pill/day for 12 weeks, double-blind study	10 mg/day	Normal liver function, no gastrointestinal effects quantified	NOAEL: 10 mg/day	Pratt et al. (1985)
Men (*n* = 24)	(1) CuSO_4_; (2) Cu glycine chelates	Daily supplements for 2 weeks each; 3 mg Cu/day as (1) 3 mg Cu/day form (1); 3 mg Cu/day as form (2); and 6 mg Cu/day as form	3.6 mg	No effects on liver or genetic damage	6 mg/day	O’Connor et al. (2003)
Men (*n* = 9)	Cu supplement	Daily intake for 147 days	7 mg/day	Plasma Cu not affected, increased Cu retention and Cu-related enzyme activity, possible immune effects	LOAEL: 7 mg/day in addition to 1.6 mg/day diet	Turnlund et al. (2004, 2005)
Men (*n* = 1)	Copper tablet, form not provided	Daily intake for 2 years of tablet, followed by higher dose for additional but unspecified time period	30 mg/day for 2 years, 60 mg/day for additional time	Acute liver failure	LOAEL: 30 mg/day	ECI (2008)
Infants (*n* = 148)	CuSO_4_	Formula-fed or breast-fed between 3 and 12 months	<0.1 or 2 (water)	No acute or chronic effects at 2 mg/L	No effect level: 2 mg/L	Olivares et al. (1998)

Please see Supplementary Table S2 for additional detail on these studies

Other studies have investigated gastrointestinal and liver effects of copper on adults such as nausea, vomiting, and liver enzyme function. The study by Pizarro et al. (1999) consisted of 60 adult Chilean women orally ingesting 0, 1, 3, or 5 mg Cu/L (prepared with CuSO_4_) for 2 weeks using a “Latin-square” design. Women prepared water at home with the specified dose and logged total amount of water consumed at the end of each day. The 2-week exposure was followed by 1 week of no exposure. In addition to assessing gastrointestinal symptoms, blood samples were analyzed for serum copper and ceruloplasmin levels and liver function via enzymatic activity, which showed no changes after the 2 weeks of exposure. The incidence of gastrointestinal symptoms was 5% (0 mg Cu/L), 8% (1 mg Cu/L), 23% (3 mg Cu/L), and 22% (5 mg Cu/L). Acute gastrointestinal effects in women were observed at 3 mg Cu/L in drinking water, with an estimated NOAEL of 2 mg Cu/L drinking water (Pizarro et al. 1999).

In a second study (Pizzaro et al. 2001), 45 adult Chilean women ingested 5 mg/L of total copper with different ratios of soluble (CuSO_4_) to insoluble (copper (II) oxide) (0:5, 1:4, 2:3, 3:2, and 5:0) for 1 week followed by a 1-week break, repeating for a total of 9 weeks. The study investigated the same effects as in Pizarro et al. (1999) (liver, gastrointestinal effects, and Cu homeostasis). The various ratios of copper forms did not affect gastrointestinal symptoms with similar symptoms observed for all ratios. Acute gastrointestinal symptoms were caused by both soluble and insoluble copper, indicating that, regardless of the ingested form, ionic copper is formed in the stomach, thereby resulting in irritation. This information seems to contradict reported differences in copper bioavailability between soluble and insoluble or less soluble forms (section 2.0 in the Supplementary Information); however, other factors likely influence dissolution rate and, hence, gastrointestinal bioavailability for copper in soil should be considered (Intawongse and Dean 2006).

In Araya et al. (2001), 179 men and women from the Unites States, the United Kingdom, and Chile ingested 0, 2, 4, 6, or 8 mg Cu/L in a single, weekly bolus dose of deionized water over 5 weeks prepared with CuSO_4_. Gastrointestinal effects were self-reported 15 min, 1, and 24 h after administration. The LOAEL was determined at 6 mg Cu/L, and the NOAEL for gastrointestinal effects and nausea was 4 mg Cu/L. Nausea was the first and most common symptom reported in the study.

In a larger multinational study by Araya et al. 2003b, 1365 adults (study centers in the Unites States, Ireland, Chile, and China; *N* = 58–73/center) used tap water daily over 2 months with 0.01, 2, 4, or 6 mg Cu/L in a double-blind study. Daily usage and ingestion included drinking and preparation of soups, foods, and/or beverages. Gastrointestinal effects were self-reported daily, and field workers visited homes every other day to consult with participants. After 2 months, blood samples were drawn from 60 participants from each experimental group for measurement of copper status (serum copper concentration, ceruloplasmin concentration, and copper-containing enzyme [superoxide dismutase] activity). Gastrointestinal effects were reported for all groups including the control, as in other studies that might be attributed to background symptoms (Araya et al. 2001, 2003a; Pizarro et al. 1999, 2001). A statistically significant increase in gastrointestinal symptoms was identified at 6 mg Cu/L with the NOAEL reported as 4 mg Cu/L. Changes in serum copper concentration or liver enzyme levels were not detectable, indicating copper homeostasis and liver function were not affected, consistent with low likelihood of liver toxicosis for most people at these elevated water copper levels in addition to background dietary copper.

Studies from Araya et al. (2001, 2003b), Olivares et al. (1998, 2001), and Pizarro et al. (1999, 2001) helped determine the dose-response relationship between copper in drinking water and acute gastrointestinal effects (WHO 2004). Based on these new studies the WHO upgraded their provisional guideline of 2 mg/L to an established guideline due to the reduction in dose-response uncertainties (WHO 2004). The 2 mg/L value is based on consumption of 2–3 L of water per day, the use of dietary supplements, and copper ingestion from food (WHO 2004) without exceeding the tolerable upper intake level of 10 mg Cu/day (IOM 2001) or leading to gastrointestinal effects (WHO 2004).

Effects of copper dose on internal copper regulation and possible systemic effects have been assessed in a few experimental studies of human subjects. Turnlund et al. (2004, 2005) evaluated copper status, copper-related enzyme levels, and immune system parameters of nine men (ages 26–49 years old) who consumed a diet with 1.6 mg Cu/day for 18 days (0.02 mg Cu/kg BW/day based on 75 kg average body weight of subjects), followed by an uncontrolled diet and copper supplements at 7 mg Cu/day (0.093 mg Cu/kg BW/day) for 129 days, and finally 18 days of 6.2 mg Cu/day supplement and a 1.6 mg Cu/day diet (0.1 mg Cu/kw BW/day). Calculated copper retention (based on absorption and excretion of radiolabeled copper) was near zero at 1.6 mg Cu/day diet intake but retention increased to 0.67 mg Cu/day after the 6.2 mg Cu/day supplement period. Plasma copper was not affected by the higher copper supplementation, but activity of ceruloplasmin, benzylamine oxidase, and superoxide dismutase increased (Turnlund et al. 2004). Some changes were also observed in the composition of white cell counts, although total white cell numbers were not affected. Antibody titers of three influenza strains were lower after vaccination in the copper-exposed subjects compared with 10 control subjects; however, large variation in these levels resulted in statistical significance for titers of only one of the strains. Other changes in measures of immune parameters or responses were not statistically significant. These results may also have been affected by the multiple comparisons being tested and the small subject numbers.

Understanding whether copper supplements increase copper exposure sufficiently to cause adverse effects is important. The primary reported effect of chronic elevated copper intake is liver damage (IOM 2001). To understand copper supplementation and its effects on the liver, a double-blind repeated crossover trial with 24 subjects was used for a total of 6 weeks (O’Connor et al. 2003). A total of three supplements were each given for 2 weeks for a total of 6 weeks in the following order and form: 3 mg Cu/day in the form of CuSO_4_ (divalent form), 3 mg Cu/day as Cu glycine chelates (divalent form), and 6 mg Cu/day as Cu glycine chelates. A modified Comet assay and assessment of liver enzymes found no significant alteration of the liver or DNA from copper supplementation, even at an ingestion of 6 mg Cu/day every day for 6 weeks, or at approximately six times the normal intake (O’Connor et al. 2003).

The study used by IOM to develop the tolerable upper intake level selected liver toxicity as the endpoint with a total of seven human test subjects using a double-blind study with 10 mg Cu/day as copper gluconate (divalent form) or a placebo for 12 weeks (Pratt et al. 1985). No liver damage or gastrointestinal effects were reported. Overall, although no liver toxicity occurred with these repeated-dose studies of copper supplements, the few studies and small study sample sizes limit conclusions about the exact conditions (dose, length of exposure, form of copper, and individual susceptibility) under which liver damage might or might not occur.

#### Toxicity studies in animals

A number of repeated-dose studies have been conducted in animals, generally at doses well above those associated with acute nausea in humans from drinking water. The animal studies provide additional information on subchronic and chronic toxicity that is generally less available in human case and experimental studies. The two-animal repeated-dose studies summarized below are considered the most informative repeated exposure studies due to the robust, guideline-compliant study designs (ECI 2008). Additional details on these studies, as well as other animal repeated-dose studies, are presented in Tables S2 and S4, and section 4.0 of the Supplementary Information.

The US National Toxicology Program (NTP) conducted 2-week dietary and drinking water exposure studies and 13-week dietary studies with CuSO_4_^.^in F344/N rats and B6C3F_1_ mice (Hébert 1993). In the 2-week NTP drinking water exposure study, rats (5/sex/group) were orally administered drinking water with copper sulfate (CuSO_4_) corresponding to copper concentrations of 76, 254, 762, 2543, or 7629 mg Cu/L. The estimated exposure levels were 10, 29, 45, 36, and 97 mg Cu/kg BW/day for males and 10, 26, 31, 31, and 71 mg Cu/kg BW/day for females). Dose groups at or above 45 mg Cu/kg BW/day for males and 31 mg Cu/kg BW/day (762 mg Cu/L) for females experienced numerous effects such as death and organ weight loss. Additional effects consistent with dehydration from reduced water consumption were also reported. An identical drinking water study was performed in B6C3F_1_ mice and results between the two studies were consistent. Equivalent toxicity in mice was observed at 58 and 62 mg Cu/kg BW/day (762 mg Cu/L) in male and female mice.

In the 2-week NTP feeding study, Fischer rats were orally administered 255, 509, 1018, 2036, or 4072 mg Cu/kg diet^4^ (equivalent to 23.4, 45.8, 92.4, 197.8, and 324.5 mg Cu/kg BW/day for males and 22.7, 44.3, 93.4, 195.7, and 285.3 mg Cu/kg BW/day for females) (Hébert et al. 1993; Hébert 1993). Feed was available to the rats for 15 days. At ≥197.8 mg CuSO_4_/kg BW/day for males and ≥195.7 mg CuSO_4_/kg BW/day for females, body weight reductions between 66 and 92% in male and female rats occurred due to a reduction in food consumption in addition to direct toxicity. At 509 mg Cu/kg diet, histopathological changes to the forestomach were observed, and hepatic lesions were noted at the two highest doses. Cell depletion in bone marrow and spleen occurred at ≥197.8 mg CuSO_4_/kg BW/day for males and ≥195.7 mg CuSO_4_/kg BW/day for females, respectively. No deaths occurred. Inflammation of the liver was noted in male rats above 197.8 mg CuSO_4_/kg BW/day. An identical study was performed for B6C3F1 mice. The NOAEL for forestomach lesions was determined at 23.4 and 22.7 mg Cu/kg BW/day for male and female rats and at 42.8 and 53.5 mg Cu/kg BW/day for male and female mice (Hébert et al. 1993; Hébert 1993).

The 13-week NTP feeding study in rats used diet concentrations of 127, 255, 509, 1018, and 2036 mg Cu/kg feed (8.1, 16.3, 32.8, 65.9, and 140.2 mg Cu/kg BW/day for males and 8.7, 17.3, 34.4, 68.0, and 134.4 mg Cu/kg BW/day for females) (Hébert 1993). No exposure-related deaths occurred. Weight loss was significant at the two highest doses. Effects at ≥509 mg Cu/kg feed included decreases in cell volume and hemoglobin, increases in enzymatic activity, and forestomach, liver, and kidney lesions. A significant dose-related increase of copper concentration occurred in the liver and kidney over the dose range. The NOAEL for forestomach, kidney, and liver effects in rats was determined to be 16.3 mg Cu/kg BW/day for male rats and the NOAEL for forestomach lesions for female rats was 17.3 mg Cu/kg BW/day. An identical study was performed in B6C3F_1_ mice, though no liver or kidney effects were observed (Hébert 1993). The NOAEL for forestomach lesions in mice was 97.2 and 125.7 mg Cu/kg BW/day for males and females, respectively. In general, liver effects in rats were only seen at doses >16.3 mg Cu/kg BW/day.

In a chronic copper feeding study in C57BL/6J mice, 1-month-old mice were given 317 mg Cu/L as copper gluconate in drinking water until various ages, ranging between 31 and 700 days (Massie and Aiello 1984). Mean survival was reduced by 14.4% and the maximum lifespan was reduced by 12.8% (Massie and Aiello 1984). In general, the NOAEL dose was the same for both gastrointestinal effects and internal organ effects (e.g., liver) in the more sensitive species, rats. Other repeated-dose studies are summarized in Supplementary Table S2 and in the review of reproductive and developmental toxicity studies below.

### Investigation of Liver Toxicosis in Populations Exposed to Elevated Copper

Studies of Indian childhood cirrhosis (ICC) and Tyrolean infantile cirrhosis are the primary reported examples of childhood liver disease resulting from exposure to high copper concentrations along with suspected genetic susceptibility (NRC 2000). In certain regions in India, liver cirrhosis occurred in some infants and young children at a time when milk was stored in copper or brass containers (NRC 2000; Uauy et al. 2008; Nayak and Chitale 2013). High copper concentrations found in the livers of children were quickly reversed with a reduction in exposure and treatment. Clinical features and symptoms of the disease, however, differed from those of Wilson’s disease patients. Moreover, age-matched asymptomatic siblings of ICC cases and control children also showed increases in copper and copper-binding proteins in liver tissue but without the structural and functional changes observed in the ICC cases. With the elimination of storing milk in materials containing copper, the disease frequency decreased, implicating copper in the etiology of the disease, although the association has been noted as circumstantial (Sriramachari and Nayak 2008). It is thought that susceptibility was related to an autosomal recessive trait because some siblings were also affected, whereas the parents of affected children were not affected in childhood (NRC 2000). However, examination of the pedigree of families with ICC and age-matched controls revealed that genetic susceptibility is likely based on multiple factors, rather than a single factor, as no clear evidence of autosomal recessive, partial sex linkage, or double recessive traits was found (Naya and Chitale 2013). No clear documentation exists for the amount of copper infants were exposed to in the Indian childhood liver toxicosis case study; however, one study tried to replicate copper concentrations potentially available through the use of brass vessels for milk storage and found up to 6.21 mg Cu/L in milk after 6 h (O’Neill and Tanner 1989), or 0.93 ± 0.1 mg Cu/kg BW/day for an exposure of a child consuming milk at a rate of 150 mL/kg BW/day.

Further investigation of the origin of the disease by a multicenter study in India indicated that copper may not be the cause in all ICC cases and that excessive liver copper may also be the result of hepatic injury from some other factors (Nayak and Chitale 2013; Sriramachari and Nayak 2008). This collaborative study across six research centers was conducted in the 1980s to clarify the cause of ICC (Nayak and Chitale 2013; Sriramachari and Nayak 2008). Based on a review of 885 children with a liver biopsy, 227 cases of definite ICC were compared with 426 non-ICC control cases for use of “copper yielding utensils.” In two research centers, none of the ICC cases used these utensils, compared with less than 2% of controls. In three research centers, use of these utensils in study subjects was 15–20% with no difference between ICC cases and controls. The remaining research center reported greater utensil use that was not statistically different between ICC cases (55%) and controls (52%). About 10% of definite ICC cases had no possible prior source of excess copper exposure. Asymptomatic siblings with some accumulation of copper in their liver (although not as high as ICC cases) showed no evidence of toxicity and had a decline in copper to normal levels after removal of copper-containing utensils. Hepatic liver concentrations increased in the later stages of ICC rather than in the early stages, suggesting, along with the lack of evidence of higher exogenous copper exposure in most ICC cases, that copper accumulation was a result rather than the cause of the disease. More likely etiologies for ICC were thought to be poor diet quality or postpartum herbal supplements given to new mothers and infants that resulted in adverse effects on the liver rather than toxicity from excess exogenous copper exposure (Nayak and Chitale 2013; Sriramachari and Nayak 2008).

In Tyrolean infantile cirrhosis, elevated copper exposure through heating milk in copper pots or the use of copper utensils was associated with liver cirrhosis and 138 pediatric deaths in an area of western Austria between 1900 and 1980 (NRC 2000; Müller et al. 1996; Uauy et al. 2008). The symptoms were indistinguishable from ICC. Liver cirrhosis appeared to run in families, although not all children within a family were affected. Both affected and unaffected siblings consumed the same milk, which was retrospectively estimated to have a copper concentration of 10.5–63.3 mg Cu/L when prepared and boiled for 20 min in brass and copper pots (Müller et al. 1996). By comparison, reported copper concentrations in breast milk range from ~0.2–1 mg Cu/L and concentrations appear to be highest soon after birth followed by a decline during the first year (ATSDR 2004). The siblings without liver toxicosis were unaffected at doses estimated to be 8–49 times higher than the current 1.3 mg Cu/L EPA action level for drinking water.

Other studies have examined whether populations with higher copper levels in drinking water have resulted in liver toxicosis in infants and children. One study was conducted in three towns in Massachusetts from 1969–1991 with 8.5–8.8 mg Cu/L in drinking water (Scheinberg and Sternlieb 1994). A total of 2788 children under the age of 6 were followed during the course of 23 years to determine the total number of infantile deaths and infantile deaths relating to liver disease. A total of 135 deaths occurred but none were attributed to cirrhosis or any type of liver disease, despite drinking water levels that were higher than the 1.3 mg Cu/L EPA action level by 6.5–6.8 times.

A study in Berlin, Germany, tested water samples from 2944 households with infants (Zietz et al. 2003). A subset of 541 infants consuming tap water with composite water concentrations in their household above 0.8 mg Cu/L (maximum 4.2 mg Cu/L) were selected for medical examination. Nearly all of these 541 infants were examined, none of whom was diagnosed with liver disease. A subset (*N* = 183) also received an analysis of serum copper concentration, liver enzyme levels, total bilirubin, and ceruloplasmin, the results of which were not associated with copper exposure. Another case study in Germany indicated that symptoms in 22 children exposed to drinking water containing 0.4–15.5 mg Cu/L were attributed to liver toxicosis, though the water concentrations were not measured until a few months after symptoms were observed (IPCS 1998), and, as for ICC, cases of liver toxicosis are not necessarily associated with copper exposure. Of the 22 children, 13 fatalities were recorded, though fatalities could not be linked to a specific copper concentration in water causing the liver toxicosis. Other cases of idiopathic copper toxicosis have occurred in Mexico (Cabrera-Muñoz et al. 2010) and in Japan (Hayashi et al. 2012).

Brewer (2000) postulated an underlying basis for genetic susceptibility to excess copper exposure may exist among those who are heterozygous for Wilson’s disease. In particular, Brewer (2000) noted that a few of these individuals show signs of alterations in copper metabolism that appear to approach those with Wilson’s disease. These individuals are not affected at normal copper exposures but are hypothesized to be more sensitive to excess copper accumulation as exposures increase. Although Wilson’s disease is relatively rare (1 in 50,000 or 1 in 100,000 live births), an estimated 1–2% of the worldwide human population is a heterozygous carrier of this disease (Das and Ray 2006; Gromadzka et al. 2010; Gollan and Gollan 1998). Gromadzka et al. (2010) examined copper metabolism and function parameters in 68 heterozygous carriers of Wilson’s disease and 31 control individuals. Heterozygote carriers had parameters largely within normal limits with three individuals showing ceruloplasmin levels that were slightly lower than the reference level. As a group, ceruloplasmin levels of the heterozygotes were 75% of the control group, but serum copper levels and urinary copper excretion were not statistically significantly different (Fig. 1 in Gromadzka et al. 2010). Gromadzka et al. (2010) also noted that internal regulation of copper depends on a number of other genetically determined regulatory factors and that any combination of regulatory factors may compensate in deficiencies associated with Wilson’s disease heterozygosity. No additional research has emerged to further evaluate the hypothesis of environmental sensitivity to copper by heterozygote carriers of Wilson’s disease, nor have additional cases of pediatric liver toxicosis with elevated copper exposure been published, despite the common use of copper in consumer products and the greater prevalence of Wilson’s disease heterozygotes than for Wilson’s disease patients.

Concerns for potential susceptibility to liver toxicosis at elevated copper levels led the NRC to recommend that the 1.3 mg Cu/L EPA action level for copper not be increased (NRC 2000). The scientific literature since does not provide additional evidence to better define a level at which liver toxicosis might occur in susceptible subgroups, although the majority of the evidence involves copper levels in water or milk well in excess of the NOAEL levels defined for acute gastrointestinal effects (i.e., 4 mg Cu/L). Exposure to excess copper is most likely through the use of copper piping for drinking water used in the public water supply and in homes (Uauy et al. 2008).

### Reproductive Toxicity

For both reproductive and developmental effects of copper, animal studies indicate that effects to offspring occur at doses that also cause maternal toxicity. Information on potential reproductive toxicity of copper derives primarily from a two-generation rat reproductive toxicity study conducted with CuSO_4_ (ECI 2008). The source study is unpublished but was undertaken for a European Regulation on Registration, Evaluation, Authorization, and Restriction of Chemicals submission and is summarized in detail in ECI (2008). The study was conducted under good laboratory practices and in accordance with standard Organization for Economic Co-operation and Development (OECD) test guidelines. Parental generation (P1) male and female Sprague–Dawley rats (30/sex/group) were fed diets containing 0, 25.4, 127, 254, and 381 mg Cu/kg for premating P1 males and 0, 1.92, 9.6, 19.1, and 29.5 mg CuSO_4_/kg BW/day for premating P1 females for at least 70 days before mating and, for females, continuing through gestation and weaning at postpartum day 21. Male and female rats from F1 litters (30/sex/group) were randomly selected to continue with dietary treatment until mating and, for F1 females, through gestation and weaning of F2 pups. Clinical observations, body weight, and food consumption were assessed at least weekly throughout the study. The study evaluated sperm quality and quantity, estrous cyclicity, mating, fertility, and implantation parameters, pup survival and development, sex ratio, sexual development, weight and gross pathology of reproductive and other organs, and histopathology of liver, brain, and reproductive organs. No treatment-related effects on any reproductive or developmental parameters were observed in any dose group for any generation. Thus, the NOAEL for reproductive toxicity was the highest dose group of 381 mg Cu/kg (reported as a copper intake of 23.6−55.7 mg Cu/kg BW/day). The ECI (2008) summary notes decreased spleen weight for P1 females and F1 and F2 male and female weanlings in the highest dose group. Although not a reproductive effect, ECI (2008) identifies this as a treatment-related adverse effect with a NOAEL of 254 mg Cu/kg (15.2–26.7 mg Cu/kg BW/day). The ECI (2008) summary of the unpublished study indicated that apparent changes in spleen weight were small (9–15%) and not statistically significant for F1 weanlings, and potentially transient (spleen weight did not differ from controls in F1 adults). Rat spleen weight can be variable; however, the study authors also showed that F1 and F2 weanling spleen weights in this study were similar to historical controls for the laboratory.

The subchronic exposure study conducted by NTP provides additional information relevant to reproductive toxicity (Hébert 1993). In that study, the highest dietary Cu doses at 68 mg Cu/kg BW/day for rats and 536 mg Cu/kg BW/day for mice did not affect male reproductive organ weights, spermatid or spermatozoal measurements, or estrous cyclicity. Other reproductive endpoints were not measured.

No human studies were identified that adequately evaluate copper exposure and reproductive endpoints. ECI (2008) cites two case-control studies that reported no association between drinking water copper concentrations and pregnancy outcomes, including spontaneous abortions, stillbirths, neonatal mortality, and congenital abnormalities (Aschengrau et al. 1989, 1993). Copper was one among many chemicals evaluated, and no data were available on individual exposures; samples were collected from the public water system, not individual taps. Thus, these studies have limited value for evaluating copper exposure.

### Developmental Toxicity

The developmental toxicity study considered to have the most rigorous design is an unpublished report of a one-generation prenatal developmental toxicity study on rabbits with copper hydroxide (details summarized in ECI 2008), conducted following OECD test method 414. This study administered 0, 6, 9, and 18 mg Cu/kg BW/day by twice daily oral gavage to pregnant female New Zealand white rabbits (22/group) on gestational days 7 through 28. Maternal toxicity occurred in both the 9 and 18 mg Cu/kg BW/day dose groups, including weight loss and reduced food intake early in the treatment period. Although some recovery occurred at the end of the study, maternal body weight gain was 31 and 72% lower than controls in the mid- and high-dose groups, respectively, and food consumption was reduced 17 and 30% in the mid- and high-dose groups, respectively. In addition, three deaths and two abortions occurred in the high-dose group, possibly due to hemorrhages or ulcerative damage to the stomach lining observed in these three animals. The appetite suppression and associated weight loss were considered local effects, similar to acute gastrointestinal effects reported in human studies. No treatment-related developmental effects were observed below the maternally toxic doses. Treatment-related visceral abnormalities or skeletal malformations were not observed at any dose level. The incidence of delayed ossification of the skull and pelvis was increased slightly in the high-dose group, and an increased incidence of supernumerary ribs occurred in the mid- and high-dose groups. The skeletal variation findings should be considered secondary to maternal toxicity. Of note, a high incidence of extra ribs was reported for all groups (64, 67, 80, and 87% incidence at 0, 6, 9 and 18 mg Cu/kg BW/day, respectively), with the higher incidence at the two highest doses likely related to maternal toxicity-induced stress. Likewise, delayed skull and pelvic ossification may be a transient state and likely represent a stress response to general maternal toxicity, as has been reported for other skeletal development delays (e.g., delayed ossification, wavy rigs, bent long bones, and bent scapulae) (Carney and Kimmel 2007; Kimmel et al. 2014). The NOAEL for maternal toxicity in this study was 6 mg Cu/kg BW/day with the decreased food consumption and associated decrease in weight gain likely related to the acute gastrointestinal effects of copper from the gavage administration of large, focused, bolus doses of copper.

Several studies in young animals have examined the potential for possible increased susceptibility to liver toxicosis at early life stages because of developing copper regulatory mechanisms (Araya et al. 2005; Bauerly et al. 2005; Fuentealba et al. 2000). Bauerly et al. (2005) administered daily doses of 0, 0.01, and 0.025 mg Cu/day in a 10% sucrose solution by oral gavage to suckling Sprague–Dawley rat pups followed by weaning to the same diet received by dams during gestation (supplemented to a 13 mg Cu/kg standard diet concentration). Pups from each group were sacrificed on postnatal days 10 and 20 to assess age- and dose-related differences in organ and whole body copper levels and effects on various regulatory proteins and copper transporters. The authors reported reduced absorption with increasing copper dose at both ages. Increased copper exposure did not affect body weight, serum copper levels, or ceruloplasmin levels, although increased copper exposure did increase the liver copper concentration. Older pups absorbed more copper with increased copper supplementation compared with 10-day-old pups, although older rats showed increased adaptive mechanisms as reflected by increased metallothionein with copper dose. Fuentealba et al. (2000) fed doses of 1500 mg Cu/kg via the diet to young rats from birth until 16 weeks of age and to adult rats for up to 18 weeks. Young rats accumulated more copper in the liver, showed more severe changes in the liver, and had higher serum enzyme activity when compared with adult rats, indicating that at a dose of 1500 mg Cu/kg, young rats were more susceptible to copper-induced liver injury.

Araya et al. (2005) fed infant rhesus monkeys formula with 0.6 mg Cu/L supplemented with 6 mg Cu/L (6.6 mg/L total; *N* = 5) from ages 0 to 5 months. No evidence of clinical toxicity or histological damage of the liver was noted under light microscopy, despite increases in liver copper. Some ultrastructural changes in liver cells (e.g., irregularly shaped nuclei containing condensed chromatin) under electron microscopy were observed after 1 month but were normal at 5 months. Twice as many apoptotic cells were observed at 5 months compared with the control, although the frequency of these cells was low in all animals. Araya et al. (2005) postulated that the ultrastructural changes observed might be early cellular damage; however, a major limitation of this study was the availability of only one liver biopsy result from each of the four control animals at 2 months old.

Two studies evaluating the developmental toxicity of copper in rodents provide limited information. Lecyk (1980) reported decreased litter size, decreased fetal weight, and an increase in skeletal malformations in the fetuses of pregnant C57BL and DBA mice fed diets with ~764 or 1018 mg Cu/kg beginning 1 month before conception and continuing through study termination, although no statistical analysis was conducted. The form of CuSO_4_ used was not reported, and values are approximated based on CuSO_4_·5H_2_O. No effects were reported in mice fed diets containing 127–509 mg Cu/kg. ECI (2008) estimated a copper LOAEL dose of 123 mg Cu/kg BW/day associated with the diet concentration of 764 mg Cu/kg. The study did not report on maternal parameters, so it was not possible to evaluate the potential relationship between developmental effects and maternal toxicity. Haddad et al. (1991) administered 0 or 0.158% copper acetate (equivalent to 0.05 mg Cu/L) in drinking water to Wistar rats for 7 weeks before mating and during gestation. Treatment was associated with reduced embryonic and fetal growth (decreased yolk sac diameter, crown-rump length, and somite number) and delayed skeletal ossification in multiple locations. Although maternal growth was unaffected, maternal toxicity included histopathological changes in the liver (hepatocyte degeneration, focal necrosis, and inflammatory changes) and kidney (degenerative changes in proximal convoluted tubules) typically associated with copper deposition. This study included only one dose (82 mg Cu/kg BW/day estimated by WHO 2004) with the developmental effects possibly being secondary to maternal toxicity.

Interactions with zinc may affect whether developmental effects occur in laboratory animal studies (Reinstein et al. 1984). In a factorial design experiment, female Sprague–Dawley rats were given combinations of 1, 10, 100, and 1000 mg Zn/kg diet (equivalent to 1.39, 13.9, 139.0, and 1390.0 mg Zn/kg BW/day)^5^ and 0.5, 5, 10, and 100 mg Cu/kg in the diet (equivalent to 0.70, 7.0, 13.9, and 139.0 mg Cu/kg BW/day) from the time of mating to birth of offspring. Fetal malformations were only noted for subjects with zinc-deficient diets (1 and 10 mg Zn/kg diet, or 1.39 and 13.9 mg Zn/kg BW/day), and the rate of malformations increased as the copper level increased when given with the zinc-deficient diet. It was determined that zinc has an antagonistic role in the diet when provided with copper (Reinstein et al. 1984).

### Neurological Disorders

Copper is an essential metal that has critical roles in brain development and function. Studies suggest that the dysregulation of copper and related metabolic disorders may also result in neurodegenerative diseases through copper-regulated mechanisms. Cellular respiration and free radical defense mechanisms rely on activities of the copper-requiring enzymes, such as cytochrome C oxidase and Cu, Zn-dependent superoxide dismutase 1 (SOD1). Though not entirely elucidated, copper appears to play a role in amyotrophic lateral sclerosis caused by increased free radical generation possibly linked to a gain of function in SOD1 (Zheng and Monnot 2012). While oxidative damage related to copper and other metal ions is considered an important aspect of neurodegenerative disorders like Alzheimer disease (AD), the role of copper is controversial. The dysregulation of metals such as copper, iron, and zinc has been implicated in oxidative stress and amyloid plaque formation, two common components in AD (Cheignon et al. 2018).

The majority of the knowledge related to copper dysregulation in neurodegenerative diseases stems from AD research for which AD patients display altered regulation of copper and other metals. Key areas of controversy for copper and AD have been whether: (1) excess or deficient copper exposure causes AD and associated pathogenesis, or (2) AD causes copper dysregulation and thereby excess or deficient copper states and neurodegeneration. While age and likely high lipid intake are the greatest risk factors for developing the disease, AD is multifactorial and complex, likely with many genetic and environmental contributing factors, and these issues regarding copper have yet to be resolved (Kardos et al. 2018).

The metal–ion excess hypothesis originated from (1) enriched levels of transition elements Fe, Zn, and Cu in amyloid plaque deposits (mainly consisting of the amyloid β peptide; Aβ) in AD (Atwood et al. 2018; Dong et al. 2003; Lovell et al. 1998) and (2) reports that synthetic Aβ aggregates into fibrils upon binding of copper ions (Bush et al. 1994). Copper’s role in plaque accumulation and neuroinflammation may also be influenced by its accumulation specifically in the brain capillaries (Singh et al. 2013). It has also been reported from in vitro studies with murine macrophage BV2 cells and in a mouse model that copper increases the inflammatory response in the brain, which may affect the impairment of plaque clearance (Kitazawa et al. 2016). In triple transgenic 3xTg-AD mice, chronic copper exposure at 250 mg/L (85 mg/kg BW/day^6^) accelerated not only amyloid pathology but also τ pathology in the brain (Kitazawa et al. 2009). Another study has shown an increase in amyloid-β plaques in the brains of Alzheimer’s patients related to a higher generation of radicals when amyloid-β sugars are in the presence of copper (Fica-Contreras et al. 2017).

At the same time, the disturbed bioavailability of copper resulting in deficiency is another feature of AD (Kaden et al. 2011), although the mechanisms and the causal relationships of the reduced copper availability in AD are not well understood (Kessler et al. 2006; Schafer et al. 2007; Klevay 2010; Bost et al. 2016; Bulcke et al. 2017; Li et al. 2017; Bagheri et al. 2018; Kardos et al. 2018). AD-associated alterations in metal–ion (primarily copper) homeostasis were found in all regions of AD brain tissue (Xu et al. 2017), suggesting a pan-cerebral copper deficiency in AD. Such a widespread brain-Cu deficiency may contribute to the pathogenesis by acting through the loss of enzyme functions in energy utilization and antioxidant defenses (Xu et al. 2017). Reduced copper bioavailability to the brain may be further enhanced by the accumulation of copper in AD plaques (Zheng and Monnot 2012), lipid rafts (Bagheri et al. 2018), or astrocytes (Kardos et al. 2018). Research suggesting that copper supplementation may be beneficial in AD include animal models either overexpressing amyloid precursor protein (APP) or APP in combination with other genes like presenilins and τ, or APP in APLP knockout mice. In the latter, copper levels were found to be increased in cerebral cortex and liver (White et al. 1999). Overexpression of APP resulted in significantly reduced brain copper levels in three different transgenic lines (Maynard et al. 2002; Bayer et al. 2003; Phinney et al. 2003). In APP23 mice which overexpress β-APP, supplementation with 65 mg/L Cu(II) in drinking water (administered as copper sulfate pentahydrate) increased brain copper levels, restored superoxide dismutase, lowered β-amyloid peptide levels, and reduced premature deaths (Bayer et al. 2003). Toxic-milk (txJ) mice with a mutant ATPase7b transporter favoring elevated Cu levels when crossed with single transgenic (Tg) CRND8 APP mice, at 6 months of age with 30 mg Cu/kg in the brain showed a reduced number of amyloid plaques and diminished plasma Aβ levels compared with homozygous TgCRND8 mice and to TgCRND8 APP controls (Phinney et al. 2003). Encouraged by the animal studies exhibiting a beneficial outcome of copper treatment, oral supplementation with Cu(II) was investigated in a clinical trial (Kessler et al. 2008). Based on indications of copper deficiency (i.e., lower copper and ceruloplasmin-bound copper levels in plasma of AD patients with advanced biomarkers of the disease in cerebrospinal fluid; Kessler et al. 2006), Kessler et al. (2008) studied the effect of daily exposure of up to 8 mg Cu(II) over 12 months in AD patients. Plasma copper levels declined in the placebo group, but stabilized in the Cu-treatment group. The treatment did not affect zinc or non-ceruloplasmin levels, although copper had neither a detrimental nor a beneficial effect on AD.

Conversely, several studies have reported the higher levels of total copper and non-ceruloplasmin-bound copper in the serum of AD patients compared with controls (Bagheri et al. 2018). Squitti et al. (2018) reported the higher levels of non-ceruloplasmin-bound copper and the similar levels of ceruloplasmin-bound copper in the serum of 385 AD patients compared with 336 healthy controls. Serum levels of non-ceruloplasmin-bound copper of AD patients were also similar to those of nine Wilson’s disease patients, although Wilson’s disease patients had lower levels of total copper and ceruloplasmin-bound copper than AD patients. Squitti et al. (2002) and Brewer et al. (2010b) attribute the dysregulation of copper in the brains of AD patients to an increase in the “labile” or exchangeable pool of copper in peripheral circulation as represented by non-ceruloplasmin copper. However, the exact chemical nature of this “labile” pool has remained undefined (Ackerman and Chang 2018). Ceruloplasmin is the main copper carrier in plasma (Harris et al. 1999), although many other proteins such as albumin have high bind affinity and capacity for copper and provide reserve binding capacity to keep extracellular ionic copper low. Albumin-bound copper can be delivered to cells and exchanged with other proteins such as transcuprein or with chelators (Linder et al. 2016), and, as noted in the toxicokinetics section, also contributes copper to ceruloplasmin.

As further hypothesized by Brewer (2010a, 2019), increased divalent copper (Cu(II)) absorption from drinking water or supplements is thought to contribute to this labile pool because divalent copper is not taken up in the gastrointestinal tract by the Ctr1 transporter at the apical membrane of the gastrointestinal tract epithelia cells and processed by the liver, including binding to ceruloplasmin, but instead is rapidly passively absorbed thereby contributing to the non-ceruloplasmin-bound, “labile” copper that can more freely enter the brain. Nevertheless, as noted in the toxicokinetics section, metalloreductases readily reduce Cu(II) for transport by Ctr1 and possibly by a distinct saturable transporter for gastrointestinal uptake of Cu(II) (Lee et al. 2002), and passive absorption appears to be limited even under conditions that would facilitate rapid absorption (e.g., administration of Cu(II) in liquid to participants in a fasting state; Hill et al. 1986; see the toxicokinetics section). Cu(II) also rapidly shifts to Cu(I) in the absence of oxygen (and vice versa).^7^

Direct evidence in humans consuming excess copper in drinking water do not support the hypothesis that Cu(II) in water elevates non-ceruloplasmin copper and the more “labile” pool of copper. Adult men and women (48–49 individuals per dose group) drinking <0.01, 2, 4, or 6 mg Cu/day in water for 2 months showed no differences in levels of non-ceruloplasmin copper or parameters reflecting copper loading including red blood cell copper, monocyte copper, superoxide dismutase, serum glutamic-oxaloacetic transaminase, serum glutamic-pyruvic transaminase, and serum gama-glutamyltransferase (Araya et al. 2003b). Thus, for the drinking water levels examined, no effect on non-ceruloplasmin-bound copper or copper status was found. Klevay (2010) has also noted that a higher proportion of non-ceruloplasmin-bound copper in AD patients may occur as a result of decreased ceruloplasmin levels in blood from copper deficiency.

Vascular factors such as hypertension, hypercholesterolemia, and diabetes as well as the inheritance of the epsilon4 allele of the ApoE gene are risk factors for AD (Sjögren and Blennow 2005). Thus, interactions of high fat diet and copper on the risk of AD have been a research interest. An animal model of hypercholesterolemic rabbits showed amyloid-inducing effects at very low levels of copper in drinking water (0.12 mg Cu/L) (Sparks 2004), with similar results reported in other susceptible species/strains (spontaneously hypercholesterolemic rabbits, dogs on a 4% cholesterol diet, transgenic mice with elevated brain amyloid-β; Sparks et al. 2006). Surprisingly, these findings were made only when copper sulfate was added to distilled water, and not when tap water was used in combination with high cholesterol feed (Sparks and Schreurs 2003). Potable tap water typically contains at least an amount of Cu of 0.12 mg Cu/L as copper carbonate or copper carbonate hydroxide (Kaden et al. 2011) which suggests that such effects occur only in the absence of other minerals normally present in tap water. In the rabbit model, extremely high dietary cholesterol levels of 1–2% leads to serious liver toxicity (steatosis and fibrosis; Buyssens et al. 1996; Xu et al. 1997; Schreurs 2013), thereby affecting copper regulation and homeostasis. Thus, the relevance of these findings have serious limitations as a model for human exposure to copper in tap water because of the unrealistically high cholesterol levels and likely involvement of liver impairment affecting copper regulation along with the absence of normal constituents in tap water. Measurements of liver effects, copper uptake, organ distribution, and excretion in comparison with normal diets versus high cholesterol in feed, would enable a better assessment of potential links between hypercholesterolemia and the risk to develop an Alzheimer-like pathology.

A prospective cohort study of 602 older individuals reported that copper supplements in conjunction with a high saturated and trans-fat diet was associated with cognitive decline (Morris et al. 2006). No effect was found for a high cholesterol diet, and AD was not examined. As cautioned by the authors, the multiple comparisons and potential effects of uncontrolled factors (and chance findings), limit causal conclusions. A randomized controlled trial would provide more clarity of the role of copper supplements. No follow up on the role of copper supplements on AD or cognitive decline has been published in the various large prospective cohort aging studies, although these studies have examined many other dietary nutrients (e.g., Harris 2012). In the Iowa Women’s Health Study, Musuru et al. (2011) reported an increased risk of total mortality in older women who took multivitamins, vitamin B6, folic acid, iron, magnesium, or copper, and a decreased risk for calcium. However, unlike for iron and calcium, associations for copper and risk of mortality were only statistically significant at the first follow up and not at the second and third, and no dose-response for copper and risk of mortality was found.

Similar to the use of copper supplementation to treat AD-related copper deficiency, copper chelation therapy has been proposed for AD based on theories of the effects of a labile pool of excess copper causing AD and the higher levels of serum levels of copper in AD patients in some studies. However, studies that investigated the role of APP and related APLPs in copper homeostasis strictly contradicted a proposed metal chelation based on a better understanding on the molecular level. Compounds like clioquinol, believed to deplete copper levels in the body (Cherny et al. 2001), were shown to drastically increase the intracellular copper concentration in cells (Treiber et al. 2004). Not surprisingly, Drew (2017) concluded that copper ion chelation therapy has not been effective and should be abandoned. Nevertheless, Squitti et al. (2017) commented that such therapies have not been fully tested and that a portion of AD patients may benefit. Copper chelator compounds may instead act therapeutically by changing the distribution of copper or facilitating copper uptake (Treiber et al. 2004).

Iron also plays a role in copper regulation in the brain (Monnot et al. 2011). For example, iron deficiency leads to greater copper transport across the blood–brain barrier and promotes copper overload in the central nervous system, though the blood-cerebrospinal fluid barrier is also able to remove excess copper from the cerebrospinal fluid (Monnot et al. 2011). Iron deficiency led to a 70% increase in cellular copper retention and was mediated by multiple transporters and enzymes (Ctr1, DMT1, ATOX1, and ATP7A) (Monnot et al. 2012). Some of the neurological effects could thus be related to interaction and imbalance associated with other essential elements such as iron (Bandmann et al. 2015).

Copper has also been connected to Parkinson’s disease, Huntington’s disease, and autism spectrum disorder (ASD), although these links are controversial and not well understood. Copper may play a role in the pathogenesis, or copper deficiency may be an indicator of those at risk for Parkinson’s (Bulcke et al. 2017). Copper accumulation in brains of Huntington’s disease patients has been implicated in accelerating disease progression; copper chelators and a reduction in copper in the diet was shown to delay the disease progression in animal models (Bulcke et al. 2017). There is some evidence of an association of copper and ASD. Copper levels in plasma of individuals with some forms of ASD were significantly higher than neurotypical individuals and therapy with zinc and B6 reduced copper plasma levels and some ASD symptoms (Russo and deVito 2011); however, the cause(s) of ASD is (or are) currently unknown but thought to be linked to both genetic and environmental factors.

Although copper homeostasis in the brain may be altered at various neurological disease conditions, the available experimental studies in healthy human subjects does not support the view of copper dysregulation due to environmentally relevant exposures to copper. Whether copper exposure affects the risk of AD, under what conditions, and at what levels in susceptible individuals has yet to be established. In general, it appears that copper at environmentally relevant exposures would be well handled by the large majority of the population.

## Carcinogenicity and Mutagenicity

There is no indication that copper is carcinogenic (ECI 2008). Though copper is not considered to be a carcinogen, copper may be a tumor promoter through the modulation of oxidative phosphorylation (Ishida et al. 2013).

Most mutagenicity studies have focused on CuSO_4_ or the effects of copper ions in relation to DNA damage. Currently, copper is classified as having no mutagenic effects (ECI 2008). ECI (2008) reviewed 19 studies from 1975 to 1997 that included cell and animal models. In vitro tests suggest that copper mutagenicity occurs at high, often cytotoxic doses; however, these conditions do not represent normal physiological conditions. The in vitro and in vivo mutagenicity studies are summarized in Supplementary Table S3.

Unlike conditions of in vitro tests, in vivo research indicates that free copper concentrations in the body are low, with the majority of copper bound to albumin or ceruloplasmin (ECI 2008). Inside of cells, it is estimated that less than one copper atom per cell is found as free copper (ECI 2008). Metal binding proteins, such as metallochaperones and antioxidants, occur at high concentrations inside and outside of cells and protect against effects from free ionic copper and other metals or reactive oxygen species (ECI 2008). The majority of these studies also indicate that CuSO_4_ does not cause mutagenicity in vivo (ECI 2008). Overall, high doses of CuSO_4_ are cytotoxic, but under normal physiological conditions with the ample presence of copper-binding proteins and low ionic copper, copper-induced oxidative damage to DNA is unlikely to occur.

## Dose Response

### Identification of Key Toxicity Endpoints and Populations of Concern

The key health effects for oral copper exposure are gastrointestinal irritation with acute or short-term exposure, as documented in several well-conducted human studies that have been published since the original study that forms the basis of EPA’s action level for copper in drinking water. Acute and repeated-dose studies of ingestion of copper in drinking water at 6 mg Cu/L in adults (0.14 mg Cu/kg BW/day) indicate that a subset of the general population may have gastrointestinal effects such as nausea, though no signs of copper imbalance or liver effects were noted (Pizarro et al. 1999, 2001; Araya et al. 2001, 2003a, 2003b; Uauy et al. 2008; see also Supplementary Tables S2 and S4).

Liver toxicosis from excess copper accumulation with repeated exposure is a potential concern particularly in the first year of life as biliary excretion of copper is developing at a time of potential for high intake per body weight of water used to make formula. Liver toxicosis in populations exposed to elevated copper in drinking water, however, is rare and appears to involve relatively uncommon genetic susceptibility to elevated copper exposure because of dysregulation of copper.

Elevated exposures in the cases of ICC or Tyrolean infantile cirrhosis had water or milk concentrations typically in excess of 5 mg Cu/L (O’Neill and Tanner 1989; Müller et al. 1996; Scheinberg and Sternlieb 1994). Not all children were affected in these populations. An experimental study of 3–12-month-old infants consuming formula made with water containing 2 mg Cu/L reported increased ceruloplasmin activity at 9 months of age but no other effects on gastrointestinal symptoms, biochemical parameters, liver function indicators, or growth/morbidity (Olivares et al. 1998). No effects other than possible early signs of liver cell damage which were reversible were noted in monkeys administered formula made with water containing 6.6 mg Cu/L from birth to 5 months of age (Araya et al. 2005).^8^

Acute gastrointestinal effects thus appear to occur at lower exposure levels (LOAEL 6 mg Cu/L) to copper in drinking water than systemic effects such as liver toxicosis (8.5–8.8 mg Cu/L; Scheinberg and Sternlieb 1994) that have been associated with copper exposure. Although a one-time exposure is sufficient to trigger a gastrointestinal reaction, use of the lower NOAEL drinking water concentration for increased frequency of nausea to calculate a daily dose is also protective of individuals with susceptibility for copper imbalance with higher copper exposures. At a NOAEL of 2–4 mg Cu/L (Araya et al. 2003a; Olivares et al. 1998) for acute gastrointestinal effects, the corresponding dose is 0.06–0.1 mg Cu/kg/day, assuming consumption of 2 L/day of water and a 70 kg body weight,^9^ or 0.2–0.4 mg Cu/kg/day for young child consuming 1 L/day at a 10 kg body weight. These doses are in addition to background dietary copper in diet and formula in the repeated-dose studies.

Animal studies indicate a higher NOAEL of 16–17 mg Cu/kg/day in the more sensitive animal species, rats, for forestomach lesions, weight reduction, and liver effects (13-week feeding study; Hébert 1993). Animal studies investigating reproductive and developmental effects of copper indicate effects to offspring occur at doses that also cause maternal toxicity (typically weight reduction); thus, traditional reproduction and development effects are not sensitive endpoints for copper.

### Limitations of Deriving an EPA Reference Dose

EPA typically develops an oral RfD by identifying a NOAEL dose or, in absence of a NOAEL, a LOAEL based on experimental or epidemiological studies. To account for uncertainty, additional factors are applied to lower the NOAEL or LOAEL value in deriving an RfD. An example is the application of a factor of 10 when extrapolating from studies in animal to humans (US EPA 1993). After incorporating default uncertainty factors of 10 each for animal-to-human extrapolation, variability within humans, and subchronic to chronic exposure extrapolation, oral RfDs calculated based on the available data for repeated-dose copper toxicity in animals are within dietary levels. For example, the NOAEL value from the best available animal studies (Hébert et al. 1993; Hébert 1993) would, after application of default uncertainty factors (UFs; e.g., 1000) (Supplementary Table S4; US EPA 1993), result in an RfD similar to the recommended dietary allowance for copper (0.9 mg Cu/day for adults or 0.013 mg Cu/kg/day; IOM 2004; Table 2). Use of the NOAEL from the human studies (e.g., 0.06–0.1 mg Cu/kg/day) with default UFs for within human variation or subchronic to chronic extrapolation (100) would result in RfDs of 0.0006–0.001 mg Cu/kg/day, orders of magnitude below the recommended dietary allowance.

**Table 2:** US and European oral dietary reference values for copper

Group	Age	Recommended oral dietary intake (mg Cu/day)	Tolerable upper intake level (mg Cu/day)	Recommended oral dietary intake (mg Cu/kg BW/day)	Body weight (kg)	Reference
Infant	0–12 months	0.20–0.22	ND	0.020–0.022	10	IOM (2001)
Infant	7–11 months	0.4	ND	0.04	10	EFSA (2015)
Children	1–8 years	0.34–0.44	1–3	0.034–0.044	10	IOM (2001)
Children	9–13 years	0.70	5	0.02	37.5	IOM (2001)
Children	1 to <3 years	0.7	ND	0.07	10	EFSA (2015)
Children	3 to <10 years	1	ND	0.01	10	EFSA (2015)
Boys, girls	10 to <18 years	1.3, 1.1	ND	0.02	53.9	EFSA (2015)
Adolescents	14–18 years	0.89	8	0.02	53.9	IOM (2001)
Adults, both genders	19–70+ years	0.90	10	0.013	70	IOM (2001)
Men, women	NA	1.6, 1.3	ND	0.02	70	EFSA (2015)
Women, pregnant, lactating	14–50 years	1.0, 1.3	8–10	0.014, 0.019	70	IOM (2001)
Adult, pregnant and lactating women	NA	1.5	ND	0.02	70	EFSA (2015)

Weights used to calculate recommended oral dietary intake (mg Cu/kg BW/day) from US EPA 1997

*ND* not determined

RfDs are intended to be protective of toxicity caused by excess exposure and are typically not below doses deemed essential and therefore well-tolerated (US EPA 2007). The complexity in determining an RfD for essential nutrients is that adverse effects are associated with both excess and deficient exposures. For essential metals, a tolerable upper intake level, which is the highest amount of a daily metal nutrient that can be consumed without adverse effects to the majority of the population, can also be developed, as reported by IOM (2001) for copper (Table 3). On the other hand, an upper tolerance level for a nutrient may not be protective for sensitive populations. Upper tolerance limits for copper are more applicable to dietary intake than for fluid intake, which is the medium that has been associated with the most common effects of copper (acute gastrointestinal effects) in humans. The upper tolerance limit recommended by IOM (2001) relied upon studies by Pratt et al. (1985) and O’Donohue et al. (1993) that determined a NOAEL of 10 mg Cu/day (Pratt et al. 1985). In Pratt et al. (1985), subjects received 10 mg of Cu/day as copper gluconate or a placebo for 12 weeks. Subjects in the two groups showed no statistical difference between the incidence of nausea, diarrhea, and heartburn. No changes were observed in biochemical analyses or in copper levels in serum, urine, or hair, and no liver damage occurred. In O’Donohue et al. (1993), a case study describes one adult male consuming 30 mg Cu/day via copper supplements for 2 years, followed by 60 mg Cu/day for an additional and unspecified amount of time (IOM 2001; O’Donohue et al. 1993). The patient required a liver transplant. Current international regulatory guidelines and recommendations for copper ingestion are found in Table 3.

**Table 3:** Summary of regulatory guidelines and recommendations for oral doses

Criteria Type	Location	Oral dose (mg/kg/day)	Specific application	Reference
Reference dose	Michigan	0.038	Generic criterion and screening level	Michigan Department of Environmental Quality (MDEQ) (2013)
Acute duration oral minimal risk level	USA	0.01	1–14 days of exposure	ATSDR (2004)
Intermediate duration oral minimal risk level	USA	0.01	15–365 days of exposure	ATSDR (2004)
EPA regional screening level RfD	USA	0.04	Based on EPA 1.3 mg/L action level	US EPA 2017
Reference dose	Texas	0.097	Developed based on sensitive groups	Texas Commission on Environmental Quality (TCEQ) (2014)
Recommended dietary allowance	USA and Canada	(0.9) 0.012	Adult intake	IOM (2001)
Categorical regression analysis optimal intake value	N/A	(2.6) 0.04	Adults, children	Chambers et al. (2010)
Proposed upper limit of acceptable range of dietary intake	International	(2–3) 0.03–0.04	Proposed guideline	International Program on Chemical Safety (IPCS) (1998)
Tolerable upper intake level	USA and Canada	(10) 5	Adult intake	IOM (2001)
Proposed safe upper level of dietary intake	International	(10–12) 0.14–0.17	Proposed guideline	WHO (1996)

Assuming an average water intake of 2 L/day for an adult

*BW* body weight

A different approach than the EPA RfD methodology, the categorical regression analysis, also recognizes copper essentiality in deriving a health-protective oral dose. Copper essentiality results in a U-shaped exposure-response relationship caused by both adverse effects at high and low doses. Categorical regression is a method that can integrate information from many types of copper deficiency and toxicity studies with variations in study design, dose concentration, or study organisms in developing an exposure-response curve by simultaneous modeling of both deficiency and excess toxicity dose-response relationships (Chambers et al. 2010; Krewski et al. 2010a, 2010b; Milton et al. 2017a). This approach has also been considered for another essential metal, manganese (Milton et al. 2017b).

The copper exposure-response data considered in the categorical regression analysis were from studies in humans or animals exposed to copper from food or water. These studies were selected using inclusion/exclusion criteria based on data quality and usefulness in an exposure assessment (Chambers et al. 2010). A total of 207 exposure-response data points were used for the copper excess category, and 208 data points were used in the copper deficiency category (Chambers et al. 2010). To harmonize the magnitude and types of health effects associated with data on doses from different studies, health effects for each data point were ranked in “severity” and associated with a dose and duration of exposure (Chambers et al. 2010). The categorical regression models rank the response based on varying categories of severity, rather than along a continuous or linear scale. Seven categories of severity were used for the copper data, including 0) no effect (NOAEL); (1) biological effects within the homeostatic range; (2) early signs of copper imbalance; (3) perturbations in copper metabolism; (4) gross reversible toxicity; (5) gross irreversible toxicity with some examples including mortality, reproductive effects, and changes to organ weight; and (6) death (Chambers et al. 2010). In addition, this analysis can be used to predict the probability of different severity levels based on one or more independent variables selected (e.g., dose, duration). The analysis is also able to incorporate variation in human studies and animal models, such as species, sex, age, and study design (Krewski et al. 2010b). Modeling was conducted using the CatReg software developed by US EPA (2000).

Modeling of a single U-shaped dose-response curve to predict a dose that minimized the risk of adverse effects from excess and deficiency identified an optimal intake level of 0.039 mg Cu/kg BW/day or 2.73 mg Cu/day at a 70 kg body weight (Milton et al. 2017a). This dose was based on exposure-response models for excess and deficiency defined at a severity rank of 2 (possible early signs of copper accumulation/deficiency) (Chambers et al. 2010).

## Discussion

### Summary of Current Copper Toxicity Knowledge

The available literature on copper continues to indicate that the most sensitive adverse effect of increased oral exposures in the general population is irritation of the gastrointestinal tract, causing acute nausea. Copper is not reported to be carcinogenic or mutagenic, nor are reproductive or in utero effects sensitive endpoints. Sensitivity to systemic copper toxicity to the liver, kidneys, or central nervous system with repeated exposure appears to be related to genetically based disorders in copper regulation. Known regulatory disorders resulting in copper accumulation and toxicity (e.g., Wilson’s diseases or other rare autosomal conditions) result in serious health consequences at normal exposure levels associated with nutritional requirements and thus cannot be managed with regulatory limits. Infants in the first year of life could be more susceptible to copper overload because of their developing copper excretion capabilities. Reduced excretory capability is in part balanced by nutritional requirements in this rapidly developing life stage; however, formula-fed infants may be more susceptible to liver toxicity at elevated copper exposures in drinking water. Nevertheless, elevated copper exposures in formula-fed infants in population-based (Scheinberg and Sternlieb 1994; Zietz et al. 2003) or experimental (Olivares et al. 1998; Araya et al. 2005) studies have not reported clear evidence of copper imbalance or liver toxicosis at drinking water levels of 2 mg Cu/L to 2–4 times higher. The relatively few isolated case studies of liver toxicosis in children therefore suggests the importance of genetic susceptibility to increased copper exposure.

### Evaluation of the Optimal Intake Level for Copper as an RfD

Compared with the recommended oral copper intake values used by IOM and the European Food Safety Authority described in Table 2 and additional recommended daily intake values in Table 4, the categorical regression-derived optimal intake value (0.04 mg Cu/kg BW/day) is 2–3 times higher than recommended oral dietary intakes for adults, similar to the acceptable upper limit proposed by the International Program on Chemical Safety (IPCS 1998), and is approximately two orders of magnitude lower than tolerable and safe upper dietary levels proposed by WHO, IOM, and Health Canada (Table 4). No studies published since the development of the 2010 evaluations of a categorical regression optimal level provide relevant toxicity information that affects this value.

**Table 4:** Summary of dietary copper daily intakes compared with the categorical regression optimal intake value

Approach	Type of recommendation	Oral dietary intake (mg Cu/day)	Oral dietary intake (mg Cu/kg BW/day)	Reference
Categorical regression analysis	Optimal intake	2.7	0.04	Chambers et al. 2010; Milton et al. 2017a
International Program on Chemical Safety	Acceptable upper limit	2–3	0.03–0.04	IPCS (1998)
WHO	Safe upper level	10–12	0.1–0.2	WHO (1996)
IOM	Tolerable upper intake for adults	10	0.14	IOM (2001)
IOM	Recommended dietary allowance for adults	0.9	0.013	IOM (2001)
WHO	Daily dietary average intake from food and water for adults	1–5	0.01–0.07	WHO (2004)
WHO	Average adult copper requirement	0.875	0.013	WHO (2004)
US NHANES	Average daily copper intake from food	1.0–1.1 for females, 1.2–1.6 for males	0.01–0.02 for females, 0.02 for males	USDA (1996)
Health Canada	Diet-based toxicity reference value for adults (+20 years)	9.87	0.14	Health Canada (2010)

The oral dietary intake value (mg Cu/kg BW/day) was calculated by assuming an average adult weight of 70 kg

*NHANES* National Health and Nutrition Examination Survey

The 2.7 mg Cu/day optimal dose (assuming a 70-kg body weight used by Milton et al. 2017) is also lower than the 6 mg Cu/day LOAEL for gastrointestinal effects and nausea for copper in drinking water observed in the human experimental trials, and the 4 mg Cu/day NOAEL for gastrointestinal effects and nausea after 2 months of exposure (Araya et al. 2003a, 2003b). Exposure to copper in drinking water in this study was in addition to 0.9 mg Cu/day estimated from the diet (Araya et al. 2003b). Thus, the optimal dose as an RfD appears to have sufficient margin of exposure to allow for additional background dietary intake. Ingestion of copper from the diet plus supplements may add 2 mg/day (IOM 2001) or about 0.03 mg Cu/kg BW/day. However, diet and multivitamin supplements would also contain other minerals such as zinc and iron that reduce copper absorption and toxicity (ATSDR 2004). The RfD is not intended to be protective of individuals with excessive ingestion of copper supplements, as demonstrated by an extreme case of liver failure resulting from consumption of a tablet with 30 mg Cu/day for 2 years followed by 60 mg Cu/day for an unknown additional duration (O’Donohue et al. 1993).

For children, the 0.04 mg Cu/kg BW/day optimal dose would be associated with a water or milk concentration for a 10-kg young child drinking 1 L/day of 0.4 mg Cu/L. This concentration is below the 1.3 mg Cu/L recommended by NRC (2000) for the protection of infants with genetic susceptibility to increased environmental copper exposure, the 2 mg/L that was a NOAEL for formula-fed infants (Olivares et al. 1998), and the copper milk concentrations associated with ICC and Tyrolean infantile cirrhosis (O’Neill and Tanner 1989; Müller et al. 1996). Similar to the studies in adults, the infants in the experimental study by Olivares et al. (1998) were also exposed to background levels of copper in formula in addition to that added from the use of drinking water to make the formula.

The optimal level (rounded to 0.04 mg Cu/kg/BW/day) based on the categorical regression is equivalent to exposure from chronic consumption (i.e., 2 L/day at a lifetime average body weight of 70 kg) of drinking water containing copper at EPA’s action level of 1.3 mg Cu/L. Accordingly, this dose (0.04 mg Cu/kg/BW/day) is also consistent with the RfD for copper (calculated from the drinking water action level) that is used in EPA’s screening level for soil and tap water (US EPA 2017). Thus, although the current literature on copper is insufficient to establish chronic toxicity levels for genetically susceptible individuals, the optimal level is also consistent with the findings of the NRC (2000) that 1.3 mg Cu/L would be protective of individuals who might be susceptible to copper metabolism disorders at higher copper concentrations. The categorical regression analysis included studies with values taken from food and water ingestion, though it is unknown whether the categorical regression analysis included copper supplement studies.

The Agency for Toxic Substances and Disease Registry (ATSDR) intermediate minimal risk level of 0.01 mg/kg/day for copper (ATSDR 2004) was based on the 2-month experimental drinking water study of Araya et al. (2003b). ATSDR, however, disagreed with the statistical tests (i.e., use of the Bonferroni multiple comparisons correction) in this study and selected a lower LOAEL at 4 mg Cu/L and NOAEL at 2 mg Cu/L. Based on the reported dose per body weight in a subset of volunteers, the corresponding dose at the NOAEL was 0.045 mg/kg/day. ATSDR divided by a UF of 3 for human variability, resulting in the minimal risk level of 0.01 mg/kg/day. This additional UF seems unnecessary given the large number of study subjects (327–355 per exposure group for gastrointestinal effects; 48–49 per group for clinical parameters), the direct nature of the effect (i.e., irritation should be less variable than for effects involving chemical metabolism, for example), the mild increase in frequency of gastrointestinal symptoms at the LOAEL (difference of 3% between 2 and 4 mg/L for the fraction reporting one or more symptoms over the 2-month period), and consistent findings of no effects at 2 mg/L and higher in several other controlled experimental human studies in other populations.

Comparisons can be made between the recommended daily copper intakes (Table 3) and regulations for copper in drinking water (Table 5). The State of Michigan (MDEQ 2015) proposed an oral RfD of 0.001 mg Cu/kg/day by dividing the ATSDR intermediate minimal risk level by a UF of 10 to extrapolate to longer-term exposure^10^. However, this extrapolation is unnecessary because the sensitive endpoint is gastrointestinal effects, which is a short-term effect, and for systemic effects the most susceptible population would be early life stages when copper regulation is less competent (OEHHA 2008). As noted by NRC (2000), the weight of evidence from the literature indicates rare occurrences of liver toxicosis are associated with genetically susceptible infants and children ingesting copper fluid concentrations well in excess of 1.3 mg/L (equivalent to 0.13 mg Cu/kg/day for 1 liter/day and 10 kg body weight).

**Table 5:** Summary of regulatory guidelines and recommendations for copper in drinking water

Criteria type	Agency	Location	Cu Water Concentration (mg/L)	Specific Application	Reference
Public Health Goal	OEHHA	California	0.3	Developed based on sensitive groups	OEHHA (2008)
Allowable Level	US Food & Drug Administration	USA	1	Bottled water	US Food & Drug Administration 21 CFR 165.110
Drinking Water Quality Standard	Ministry of Health, Labor and Welfare	Japan	1	Part of waterworks law established in 1958	Ministry of Health, Labor and Welfare (2004)
Australian Drinking Water/Aesthetics Guideline	NHMRC	Australia	1	endorsed 2001	NHMRC (2011)
Proposed aesthetic objective	Health Canada	Canada	1.0	Proposed	Health Canada (2018)
Lead and Copper Rule	US EPA	USA	1.3	Action Level and MCLG	US EPA 56 FR 26460-26564
Australian Drinking Water Guideline	NHMRC	Australia	2	endorsed 2001	NHMRC (2011)
Guideline for copper content in drinking water for population with normal copper homeostasis	WHO	International	2.0	Guideline	WHO (2004)
Proposed maximum acceptable concentrations	Health Canada	Canada	2.0	Proposed	Health Canada (2018)

*MCLG* maximum contaminant level goal, *NHMRC* National Health and Medical Research Council, *OEHHA* California Office of Environmental Health Hazard Assessment

The state of California (OEHHA 2008) has derived a public health goal (PHG) for copper in drinking water of 0.3 mg Cu/L, based on a NOAEL for gastrointestinal effects in infants of 2 mg/L from the experimental study of copper in water used to make formula (Olivares et al. 1998). OEHHA (2008) identified an equivalent NOAEL dose of 0.426 mg Cu/kg/day from water and formula, based on the highest dose reported for the high-dose infant group (mean intake plus the standard deviation) reported for the first 3 months of exposure. A UF of 3 was applied to the NOAEL to account for study uncertainties, resulting in a dose of 0.142 mg Cu/kg/day. To derive a PHG drinking water level, OEHHA applied a relative source contribution of 0.5 and used a 95th percentile infant water consumption per body weight from national survey data.^11^

The state of Texas developed an oral RfD for copper of 0.097 mg Cu/kg/day (TCEQ 2014) also based on Olivares et al. (1998). However, TCEQ used a time-weight average concentration from this study of 0.291 mg Cu/kg/day as the NOAEL, and applied a UF of 3 (due to the limited availability of human data) to derive an oral RfD (TCEQ 2014).

The Federal-Provincial-Territorial Committee on Drinking Water (Health Canada) recently proposed a maximum acceptable concentration for total copper in drinking water of 2.0 mg Cu/L (Health Canada 2018). The maximum acceptable concentration is also based on Olivares et al. (1998). A tolerable daily intake for free copper (0.426 mg Cu/kg/day total dose from water and formula) was estimated as protective of gastrointestinal effects and liver function in formula-fed infants. The average body weight of an infant of 7 kg and tap water consumption rate of 0.75 L/day for an infant as well as a relative source contribution of 0.5 were applied to the tolerable daily intake resulting in a maximum acceptable concentration of 2.0 mg Cu/L. 12Though Health Canada and OEHHA (2008) use the same NOAEL derived from Olivares et al. (1998), different recommendations are provided by these two agencies due to differences in the estimated daily volume of tap water consumed and a UF.

A copper risk assessment (summarized by Delbeke et al. 2010), initiated by the copper industry with methodologies developed in collaboration with other stakeholders, was reviewed and endorsed by both the EU Technical Committee on New and Existing Substances and the EU Scientific Committee on Health and Environmental Risks (ECI 2008). This assessment derived an internal systemic safe threshold value for workers of 0.041 mg Cu/kg BW/day based on liver effects from animal studies and assuming a human oral absorption range of 30–60% for copper. The value corresponds to an upper limit of 2.87 mg Cu/day for ingested copper, assuming a body weight of 70 kg. A safe threshold value for water of 4 mg Cu/L was also derived based on acute nausea and gastrointestinal effects in humans. The 2–3 mg Cu/day is comparable with the proposed upper limit of acceptable range of oral intake proposed by ICPS (1998) and is similar to the 2.7 mg Cu/day proposed by Milton et al. (2017a) for the general population.

In summary, most of the available criteria are similar or higher than an RfD of 0.04 mg/kg/day. The few lower criteria (i.e., ATSDR MRL) are related and are lower than necessary because of the use of additional UFs that are not warranted based on the body of evidence.

### Uncertainties in the Categorical Regression Analysis and Assessment of an RfD

Although the CatReg software is recognized as somewhat simplistic, it is still considered the most reliable method for determining a dose-response curve for essential nutrients. Research needed to strengthen the copper model developed by Krewski et al. (2010a) includes more research in humans, animal models, various life stages, and multiple endpoints, with all experiments using standard designs to better refine the categorical regression analysis technique. Historically, animal studies have focused on severely deficient copper doses, and less information is available on the details of health effects over a wide range of deficient copper concentrations in multiple target organs and other endpoints such as reproductive, cardiovascular, developmental, and immunological effects (Stern 2010). The copper literature shows that more studies on marginal excess or marginal deficiency are needed, as well as a broad range of markers used in the same studies (Krewski et al. 2010a). More information from chronic exposures was also noted as an area of research need (Krewski et al. 2010a), although based on the mode of action for copper, doses that are protective of gastrointestinal effects and that do not disrupt copper homeostasis would also be expected to be protective of long-term exposure. Although more research would help refine the model, the resulting optimal level is generally supported by the overall weight of evidence on toxicity as well as experience on well-tolerated doses within dietary intakes.

Potential limitations of the categorical regression analysis include the use of a relatively small dataset; however, over 400 exposure-response data points were used in the analysis. Another limitation is an uneven distribution of data in the various severity rankings (Chambers et al. 2010). For example, in human drinking water studies, no severity ranks between 1 and 3 were classified, with only severity in 0 (no effect) or 4 (gross reversible toxic effects) observed (Chambers et al. 2010). In other cases, severity categories were combined if no studies were found, e.g., for copper exposure studies, no severity rank of 5 was available, and therefore severity levels 4–6 were combined (Chambers et al. 2010). No deficiency studies were available in any model organism for the severity rank of 5. Running the categorical regression analysis with severity ranks grouped into a total of three categories was also tried as too many severity rank categories may not have been appropriate given the small sample size (Chambers et al. 2010).

The ability to modify the model gives some allowance for data quality or data limitations. In addition, because categorical regression considers multiple exposure media (food versus water), variations in absorption is also indirectly considered; for example, a lower dose in drinking water produced the same effect as a higher dose in food (Chambers et al. 2010). However, more information is needed for understanding the comparison between copper bioavailability in water ingestion and food intake. The available copper dataset also needed some extrapolations: human data among the higher severity ranks were estimated due to a lack of data, and short-term data were used to estimate subchronic exposure-response relationships (Chambers et al. 2010). In addition, a sparseness of data for chronic exposures made characterizing an acceptable and reliable chronic exposure range challenging (Chambers et al. 2010).

Despite its limitations, and though not specifically designed for developing an RfD, the categorical regression analysis provides information on a dose that is likely to be protective of adverse effects from excess exposure without being unreasonably low for an essential and well-tolerated element. Although the available studies and case reports indicate higher levels may also be without gastrointestinal effects or copper imbalance, uncertainties in the levels at which susceptible individuals might have copper accumulation and liver or other organ toxicity indicates caution in raising the RfD. Genetic susceptibility is likely to involve multiple genes, resulting in a dose continuum at which individuals may be susceptible to copper overload. An RfD that is well within customary tolerable levels with a considerable margin of exposure from levels known to result in imbalance in susceptible individuals should be protective of even those with environmental sensitivity. The small fraction of individuals at risk of copper overload within normally tolerated doses would likely require medical care for background exposures.

## Conclusions

Due to the essentiality of copper, assessments of oral dose-response have remained a challenge, especially given the uncertainties with genetically susceptible populations. The traditional IRIS-based RfD calculation using a NOAEL or LOAEL from animal or human studies with default UFs results in values below the recommended daily intake for copper. Categorical regression analysis provides an optimal dose with the least potential for risk of health effects from both deficiency and toxicity in humans. The resulting dose of 2.7 mg Cu/day, or 0.04 mg Cu/kg BW/day given a 70-kg lifetime average, could be considered as a chronic RfD, based on the mode of action of copper overload for which homeostatic copper regulation with subchronic exposures should be protective of long-term exposures as well. This dose is also protective of children and those with genetic susceptibility to increased copper intake (above typical levels) based on experimental, observational, or case studies of infants and children exposed to elevated copper in drinking water, formula, or milk. Using the optimal intake value as an RfD is therefore protective of both adults and children.

## Supplementary Information

Supplementary Information

## Abbreviations

AD: Alzheimer’s disease
AROI: acceptable range of oral intake
ASD: autism spectrum disorder
ATSDR: Agency for Toxic Substances and Disease Registry
BW: body weight
ECHA: European Chemical Agency
ECI: European Copper Institute
EPA: US Environmental Protection Agency
EU: European Union
GLP: Good laboratory practices
ICC: Indian childhood cirrhosis
IOM: Institute of Medicine
IRIS: Integrated Risk Information System
IUD: intrauterine devices
LOAEL: lowest-observed-adverse-effect level
MAC: maximum acceptable concentration
MCLG: maximum contaminant level goal
NOAEL: no-observed-adverse-effect level
NTP: National Toxicology Program
NRC: National Research Council
OECD: Organization for Economic Co-operation and Development
OEHHA: California Office of Environmental Health Hazard Assessment
PHG: public health goal
REACH: European Regulation on Registration, Evaluation, Authorization, and Restriction of Chemicals
RfD: reference dose
TCEQ: Texas Commission on Environmental Quality
TDI: tolerable daily intake
UF: uncertainty factor
UDS: unscheduled DNA synthesis
WHO: World Health Organization.

## Footnotes

1 DART is a database provided online by TOXNET, a toxicology data network that is sponsored by the NIH US National Library of Medicine. DART can be accessed at: https://toxnet.nlm.nih.gov/newtoxnet/dart.htm.

2 This value was approximated by using intake values for adults. A 2-L/day water intake and an average value of 0.055 mg Cu/L in tap water (0.055 mg Cu/L is the average a range of Cu found in tap water, 0.02–0.075 mg Cu [ATSDR 2004]. 0.055 mg Cu/L is multiplied by an ingestion of 1.75 mg Cu/day for food plus supplement (average between 1.3–2.2 mg Cu/day [IOM 2001]), divided by a 70-kg body weight.

3 Based on conversion in Van den Berg et al. (1994).

4 mg Cu/kg diet converted to mg Cu/kg BW/day by assuming a rat body weight of ~11 kg.

5 Average rat BW was 0.195 kg; average ingestion rate of 0.272 kg feed/day was compiled from all experimental conditions (Reinstein et al. 1984).

6 Assuming a daily water intake of 0.34 L/kg BW/day in mice, and an average weight of 0.0203 kg for adult male and female mice (US EPA 1988b).

7 https://pubchem.ncbi.nlm.nih.gov/compound/cupric_ion

8 Insufficient information was provided to calculate body weight-normalized, daily exposures.

9 From 0.048 to 0.095 mg/kg/day assuming the mean water intake (1.5 L/day) reported in Araya et al. (2003b) and an average body weight from data reported for participants in the control and 6 mg/L dose groups (63 kg).

10 The proposed RfD was never accepted. The RfD reverted to the earlier value, which is 0.038 mg/kg/day (MDEQ 2013), and is similar to the RfD used by EPA to calculate Region Screening Levels for copper.

11 [426 µg/kg BW/day × 0.5]/[3 × 0.221 L/kg-day] = 321 µg/L as the public health goal for drinking water.

12 [426 µg/kg BW/day × 7 kg × 0.5]/0.75 L/day = 1988 µg/L as the maximum acceptable concentration.
