# Correction to: Critical Review of Exposure and Effects: Implications for Setting Regulatory Health Criteria for Ingested Copper

**DOI:** 10.1007/s00267-022-01618-7

**Published:** 2022-03-11

**Authors:** Alicia A. Taylor, Joyce S. Tsuji, Michael R. Garry, Margaret E. McArdle, William L. Goodfellow, William J. Adams, Charles A. Menzie

**Affiliations:** 1 Exponent, Inc., 475 14th Street, Suite 400, Oakland, CA 94612 USA; 2 Exponent, Inc., 15375 SE 30th Place, Suite 250, Bellevue, WA 98027 USA; 3 Exponent, Inc., One Mill and Main Place, Suite 150, Maynard, MA 01754 USA; 4 Exponent, Inc., 1800 Diagonal Road, Suite 500, Alexandria, VA 22314 USA; 5 Red Cap Consulting, 7760 North Boulder Drive, Lake Point, UT 84074 USA

Correction to: Environmental Management (2020) 65:131–159

10.1007/s00267-019-01234-y

The original version of this article unfortunately contained a mistake. In Table 3 of this article, the data in the column headed Oral Dose, Specific Application, and Reference were corrected.

The below footnote and reference correlated to table 3 correction has been included in the article:

Footnote

The proposed RfD was never accepted. The RfD reverted to the earlier value, which is 0.038 mg/kg/day (MDEQ 2013), and is similar to the RfD used by EPA to calculate Region Screening Levels for copper.

The original article has been corrected.
