# Supplementary material for: Critical Review of Exposure and Effects: Implications for Setting Regulatory Health Criteria for Ingested Copper
Source: Environ Manage. 2019 Dec 12;65(1):131–59. doi: 10.1007/s00267-019-01234-y (PMC6960211; doi:10.1007/s00267-019-01234-y)
Supplement: Supplementary file 1 — Supplementary Information [file 267_2019_1234_MOESM1_ESM.docx]

Supplemental Information for

# Critical review of recent exposure and effects research: implications for setting regulatory health criteria for ingested copper

Alicia A. Taylor,^1^ Joyce S. Tsuji,^2^ Michael R. Garry,^2^ Margaret E. McArdle,^3^ William L. Goodfellow, Jr.,^4*^ William J. Adams,^5^ and Charles A. Menzie^4^

^1^ Exponent, Inc., 475 14^th^ Street, Suite 400, Oakland, CA 94612, [ataylor@exponent.com](mailto:ataylor@exponent.com)

^2^ Exponent, Inc., 15375 SE 30^th^ Place, Suite 250, Bellevue, WA 98027, [mgarry@exponent.com](mailto:mgarry@exponent.com); tsujij@exponent.com

^3^ Exponent, Inc., One Mill and Main Place, Suite 150, Maynard, MA 01754, [mmcardle@exponent.com](mailto:mmcardle@exponent.com)

^4^ Exponent, Inc., 1800 Diagonal Road, Suite 500, Alexandria, VA 22314, [wgoodfellow@exponent.com](mailto:wgoodfellow@exponent.com); [camenzie@exponent.com](mailto:camenzie@exponent.com)

^5^ Red Cap Consulting, 7760 North Boulder Drive, Lake Point, UT 84074, [adamsw10546@gmail.com](mailto:adamsw10546@gmail.com)

*Corresponding author

[wgoodfellow@exponent.com](mailto:wgoodfellow@exponent.com)

(t) (717) 793-2791

(f) (571) 227-7299

**Critical review of recent exposure and effects research: implications for setting regulatory health criteria for ingested copper**

**Table of Contents for the Supplemental Information**

1. Chemical substances, properties, and uses……………………………………………...S4
2. Bioavailability considerations……………………………………………………..……S4
3. Irritation and sensitization………………………………………………………………S6
4. Additional information on animal model repeat dose studies…………………………..S7
5. Detailed toxicological information…………………………………………………...…S8
6. References……………………………………………………………………………..S30

# Chemical substances, properties, and uses

Copper (Cu) is a transition metal. It is stable in the metallic state and has more than one oxidation state: cuprous (Cu^+^ or the monovalent cation) and cupric (Cu^2+^ or the divalent cation). A trivalent cation (Cu^3+^) occurs but is unimportant for biological systems. Cu^+^ is unstable in aqueous environments and undergoes reduction oxidation reactions to form Cu^2+^ ions or compounds or precipitates as metallic copper (Cu^0^). In water, soil, and sediment, copper binds to organic and inorganic materials with a binding affinity that is pH dependent (ATSDR 2004; ECHA 2008). For example, copper complexes with -NH_2_, -SH, and -OH groups in organic compounds such as humic acid. In the earth’s crust, copper is often found in sulphite-based minerals.

Copper has many uses due to its malleable, ductile, anti-corrosive, thermal and electrical conductivity properties (ATSDR 2004; ECHA 2008). Consumer applications are predominantly in energy and water systems such as wiring and piping in domestic water supplies, gas distribution, water heating systems, air conditioning systems, and in building materials (ATSDR 2004; ECHA 2008). Microionized copper pressure-treated lumber is also commonly used and presents a consumer exposure pathway through household products (Hristozov et al. 2018; Platten et al. 2014).

To a lesser extent, copper is used in coins, kitchen utensils, coatings (ATSDR 2004; WHO 2004), brake pads, printing inks, paints, cosmetics, and as an anti-fouling agent (ATSDR 2004; ECHA 2008; WHO 2004). Copper salts are used in agricultural applications such as bactericides, fungicides, and as an essential element in fertilizers supporting animal and plant growth (ATSDR 2004; ECHA 2008; WHO 2004).

# Bioavailability considerations

Table S-1 summarizes the bioavailability of different forms of copper. Bioavailability may in part be reflected by water solubility (Vasconcelos et al. 2007), although insufficient studies have been conducted to estimate oral bioavailability for human health risk assessment of copper compounds. Bioavailability of less soluble copper compounds in soil would be useful to quantify as has been done for metals such as lead (U.S. EPA 2007). Differences in bioavailability are much less significant for dissolved concentrations of copper in water. Both bioavailability and bioaccessibility have been reviewed in Europe as an additional method for classification of metals, but at this time methods have not been standardized for use in human health risk assessments (EBRC 2007); however, bio-elution protocols are under review by the Organization of Economic Co-operation and Development (OECD).

**Table S-1.** Summary of copper bioavailability information by copper compound

| **Compound** | **% Bioavailable** | **Water Solubility (mg/L)** | **Experimental Conditions** | **Medium** | **Reference** | **Expected Bioavailability** |
| --- | --- | --- | --- | --- | --- | --- |
| Cupric acetate | 93%** | NA | Cu liver uptake in sheep | Diet | Ledoux et al. 1995 | High |
| Copper carbonate | 54.3 ± 8.52 %** | NA | Poultry tissue accumulation study | Diet | Ledoux et al. 1991 | High |
| Copper carbonate | 121%** | NA | Cu liver uptake in sheep | Diet | Ledoux et al. 1995 | High |
| Cupric chloride | 100%** | NA | Cu liver uptake in sheep | Diet | Ledoux et al. 1995 | High |
| Copper (I) oxide | ND | >6.39 | 20°C, pH=6.6 | Not provided | ECHA 2008 | High |
| Copper oxide | 35%** | NA | Cu liver uptake in sheep | Diet | Ledoux et al. 1995 | Low |
| Copper oxide | 0.54 ± 6.86** | NA | Poultry tissue accumulation study | Diet | Ledoux et al. 1991 | Low |
| Copper (II) oxide | ND | <0.39 | 20°C, pH=6.0 | Not provided | ECHA 2008 | Low |
| Copper oxychloride | ND | >1.19 | 20°C, pH=6.6 | Not provided | ECHA 2008 | Medium |
|  |  |  |  |  |  |  |
| Copper sulfate | ND | 266,000 | Not provided | Not provided | ECHA 2008 | Soluble; high |
| Copper sulfate | 88.5 ± 11.48%** | NA | Poultry tissue accumulation study | Diet | Ledoux et al. 1991 | High |
| Copper sulfate | 142%** | NA | Cu liver uptake in sheep | Diet | Ledoux et al. 1995 | High |
| Copper powder | ND | ND | ND | ND | ECHA 2008 | Low, considered analogous to Cu (II) oxide |
| Copper methionine complex | 96% | NA | Poultry absorption study | Diet | Aoyagi and Baker 1993 | High |
| Cu | 18–25% | NA | Radio-tracing in rat diet | Breast milk | Lönnerdal et al. 1985 | High |
| Tribasic Cu chloride | 112%** | NA | Poultry feeding study | Diet | Miles et al. 1998 | High |

**Notes:**

* Dissolution tests yielded maximum releases of Cu ions <0.3 mg/L.

** Relative bioavailability.

NA is not applicable.

ND is not determined.

ECHA 2008 data based on feeding trials with pig, cattle, or chicken.

# Irritation and sensitization

While important components of the EPA’s Integrated Risk Information System (IRIS) process, irritation and sensitization are often discussed as a function of skin contact, and are less relevant for assessing risk from copper as they have little effect on the oral exposure pathway and therefore little relevance when determining drinking water-based regulations. Irritation, specifically to the skin, eye, and respiratory tract, and sensitization are briefly summarized below.

For skin, copper (I) oxide, copper (II) oxide, copper oxychloride, and copper powder currently have no classification for skin irritation (ECHA 2008). Copper sulfate (CuSO_4_) is classified as an irritant (ECHA 2008). No published data are available for skin irritation in animal studies; however, 12 unpublished animal studies show that irritation resolves by the end of the exposure (ECHA 2008). No studies have been published on skin irritation of copper or copper compounds in humans, which is unsurprising given its wide use; however, unpublished data on copper eye irritation in animals are available (ECHA 2008). Mild to moderately severe eye irritation was observed in animal studies, with some effects being reversible. Currently, copper (I) oxide, copper (II) oxide, copper oxychloride, and copper powder have no classification for eye irritation. ECHA (2008) recommends that copper (I) oxide and CuSO_4_^.^5H_2_O (copper sulfate) be classified as an irritant.

To date, copper (I) oxide, copper (II) oxide, copper oxychloride, CuSO_4_, and copper powder are not classified for sensitization (ECHA 2008). Of eight studies on skin sensitivity in animal models, three studies showed a slight skin response (ECHA 2008). There are reported cases of skin reactions in humans when exposed to copper metal from jewelry and intrauterine devices (IUDs) and from copper salts (ECHA 2008). Positive reactions for patch tests (1% of volunteers) have been reported for CuSO_4_ and copper oxide. Cross reactivity potential is possible with copper salts.

# Additional information on animal model repeat dose studies

In the 13-week NTP feeding study (Hébert 1993), Fischer rats, 10 male and 10 female per group, were orally administered CuSO_4_ in the diet at 127, 255, 509, 1,018, and 2,036 mg CuSO_4_/kg feed (8.1, 16.3, 32.8, 65.9, and 140.2 mg CuSO_4_/kg BW/day for males and 8.7, 17.3, 34.4, 68.0, and 134.4 mg CuSO_4_/kg BW/day for females) for 92 days. No exposure related deaths occurred. Weight loss was significant at 1,018 and 2,036 mg CuSO_4_/kg feed. Reduced food consumption occurred at 2,036 mg CuSO_4_/kg feed. Changes at ≥509 mg CuSO_4_/kg feed included decreases in cell volume and hemoglobin, increases in enzymatic activity, and, at 1,018 mg CuSO_4_/kg diet, iron deficiency. Forestomach, liver, and kidney had observable lesions at ≥509 mg CuSO_4_/kg feed. A dose-related increase of copper concentration occurred in the liver and kidney at all doses. The NOAEL for forestomach, kidney, and liver effects in rats was determined to be 16.3 CuSO_4_/kg BW/day for males and the NOAEL for forestomach only for females was 17.3 mg CuSO_4_/kg BW/day. Kidney effects were more noticeable in males. An identical study was performed in B6C3F_1_ mice, though no liver or kidney effects were observed. The NOAEL for forestomach lesions in mice was 97.2 and 125.7 mg CuSO_4_/kg BW/day for males and females, respectively. In general, liver effects in rats were only seen at lower doses (i.e., >16.3 mg CuSO_4_/kg BW/day) which were comparable to NOAELs for other adverse effects observed in similar toxicity studies.

# Detailed toxicological information

Detailed information on available toxicological data are provided in Tables S-2, S-3 and S-4. Animal repeated dose studies are presented in Tables S-2 and S-4, and in Section 4.0. Studies described in Table S-2 provide a broad overview of the available information on copper. Studies in Table S-4 focused on gastrointestinal and liver endpoints. Studies found in section 4.0 are considered reliable by ECHA (2008) due to their robust study designs.

**Table S-2.** Summary of available literature for repeat dose copper studies in animals and humans. Table modified from ECHA (2008).

|  |  |  |  | **Dose** | |  |  |  |  |
| --- | --- | --- | --- | --- | --- | --- | --- | --- | --- |
| **Model Organism** | **Cu Form** | **Exposure Media** | **Treatment** | **Diet or Water Concentration (ppm)^a^** | **mg/kg BW/day^b^** | **Endpoints** | **Results Summary** | **LOAEL or NOAEL** | **Reference** |
| **Animal Studies** | | | | | | | | | |
| Sprague Dawley rat, male and female | Copper sulfate | Feed | P1 male = 70 days prior to mating;  P1 females = 70 days prior to mating plus through pregnancy and until F1 weaning; F1 male = weaning through mating; F1 females = weaning through mating plus through pregnancy and until F2 weaning | 0, 25.4, 127, 254, 381 | Dependent on generation and life-cycle phase, Range: 1.53–55.7 | Spleen weight, reproductive toxicity | Multiple fertility endpoints | NOAEL (spleen growth): 15.2–26.7 mg/kg BW/day;  NOAEL (reproductive toxicity):  43.8 mg/kg BW/day (during premating);  26.5 mg/kg BW/day (during gestation);  55.7 mg/kg BW/day (during lactation) | ECHA 2008 |
| Fischer 344 rat, male | Copper sulfate | Feed | Feed for three months | 750, 1,000, 1,250, 1,500, 2,000 | 62, 82, 103, 124, 165 | Hepatic effects, parenchymal necro-inflammatory foci | Hepatotoxicity changes at all doses, histopathological changes significant >1,250 mg/kg reduced weight gain at ≥1,500 mg/kg | LOAEL: 62 mg/kg BW/day | Aburto et al. 2001 |
| B6C3F_1_ mouse, male and female | Copper sulfate | Water | Drinking water for 15 days | 76, 254, 762, 2,543, 7,629 | 10, 24, 58, N/A, 367 for male; 15, 36, 62, N/A, 330 for female | Reduced water consumption, body and organ weight, clinical toxicity symptoms (emaciation, hypoactivity, dyspnea, tremors) related to dehydration, death | Increased mortality and toxicity above 762 mg/L, effects due to decreased water intake | LOAEL: 762 mg/L | Hébert et al. 1993; Hébert 1993 |
| Fischer rat, male and female | Copper sulfate | Water | Drinking water for 15 days (5/sex/group) | 76, 254, 762, 2,543, 7,629 | 10, 29, 45, 36, 97 for male; 10, 26, 31, 31, 71 for female | Reduced water consumption, body and organ weight, clinical toxicity symptoms (emaciation, weight loss, hypoactivity, dyspnea, tremors) related to dehydration, death | Increased mortality, weight loss and toxicity above 762 mg/L, effects due to decreased water intake | LOAEL: 762 mg/L | Hébert et al. 1993; Hébert 1993 |
| B6C3F_1_ mouse, male and female | Copper sulfate | Feed | Feed for 15 days | 255, 509, 1,018, 2,036, 4,072 | 42.8, 92.1, 196.8, 293.7, 717 for male; 53.5, 103.8, 216.1, 397.8, 780.8 for female | Forestomach lesions | Forestomah Lesions | NOAEL: 42.8 and 53.5 mg/kg BW/day for male and female | Hébert et al. 1993; Hébert 1993 |
| Fischer rat, male and female | Copper sulfate | Feed | Feed for 15 days | 255, 509, 1,018, 2,036, 4,072 | 23.4, 45.8, 92.4, 197.8, 324.5 for male; 22.7, 44.3, 93.4, 195.7, 285.3 for female | Forestomach lesions, marrow marrow/spleen cell depletion | Forestomach lesions (509 mg/kg), changes to liver, kidney, marrow marrow/spleen cell depletion at 2,036 and 4,076 mg/kg in males and females, no deaths occurred | NOAEL: 23.4 and 22.7 mg/kg BW/day for male and female | Hébert et al. 1993; Hébert 1993 |
| B6C3F_1_ mouse, male and female | Copper sulfate | Feed | Feed for 13 weeks | 255, 509, 1,018, 2,036, 4,072 | 44, 97.2, 187.3, 397.8, 814.7 for male; 52.2, 125.7, 166.7, 536, 1,058 for female | Forestomach lesions | Forestomach effects | NOAEL: 97.2 and 125.7 mg/kg BW/day for male and female | Hébert et al. 1993; Hébert 1993 |
| Fischer rat, male and female | Copper sulfate | Feed | Feed for 13 weeks | 127, 255, 509, 1,018, 2,036 | 8.1, 16.3, 32.8, 65.9, 140.2 for male; 8.7, 17.3, 34.4, 68.0, 134.4 for female | Forestomach lesions, liver toxicity, kidney toxicity | Forestomach, liver, kidney effects, changes in urine chemistry | NOAEL (forestomach): 16.3 and 17.3 mg/kg BW/day for male and female; NOAEL (liver and kidney): 16.3 mg/kg BW/day for male | Hébert et al. 1993; Hébert 1993 |
| Male rat | Copper sulfate | Feed | Feed for 1–15 weeks | 2,000 | 165 | Liver and kidney toxicity | Histopathology in liver and kidney, regenerative activity after week 9 indicating tolerance | LOAEL: 165 mg/kg BW/day (only dose tested) | ECHA 2008 |
| Male rat | Copper sulfate | Feed | Feed for 1–15 weeks | 2,000 | 165 | Liver damage | Increase in copper concentration in blood and plasma, no increase in ceruloplasmin levels, increased ALT protein levels | LOAEL: 165 mg/kg BW/day (only dose tested) | ECHA 2008 |
| Male rat | Copper sulfate | Feed | Study #1: feed for up to 15 weeks; Study #2 feed for up to 15 weeks followed by second dose for three weeks | 3,000, 4,000, 5,000, 6,000; second dose when applicable: 3,000, 6,000 | 247, 330, 412, 494; second dose when applicable: 247, 494 | Hepatotoxicity | Hepatotoxicity at all doses | LOAEL: 247 mg/kg BW/day | Haywood and Loughran 1985 |
| Fischer 344 rat, male and female | Copper sulfate | Feed | feed for 18 weeks (adults), feed for up to 12 weeks (young) | 1,500 | 88 (male), 118 (female) | Liver damage | Liver damage in young and old rats, more severe in young rats, no sex difference | LOAEL: 88 and 118 mg/kg BW/day (only dose tested) | Fuentealba et al. 2000 |
| Sprague Dawley rat, male and female | Copper monochloride | Gavage | Daily for 30 days | 0, 1.3, 5, 20, 80 mg/kg/day | 0, 1.3, 5, 20, or 80 | General toxicity, reproductive, and developmental toxicity | Decrease in food consumption, red blood cells, hemoglobin, hematocrit, and proteins, increased incidence of squamous cell hyperplasia in the stomach, increased hematopoiesis in the femur, increase in runt pups | NOAEL (general toxicity): 5 and 1.3 mg/kg/day for males and females; NOAEL (reproductive/ developmental toxicity): 20 mg/kg/day | Chung et al. 2009 |
| Wistar rat, male | Copper sulfate | Gavage | Daily up to 90 days | ND | 100 or 200 | Body weight, behavior, blood proton levels | Neurobehavioral abnormality and liver and kidney dysfunction | LOAEL: 100 mg/kg BW/day | Kumar et al. 2015 |
| Wistar rat, male | Copper sulfate | Gavage | Daily up to 90 days | ND | 100 or 200 | Histopathological changes in brain, kidney, and liver | Edema, hemorrhage, necrosis, fibrosis, with highest dose over longest time having most severe symptoms such as structural damage to organs | LOAEL: 100 mg/kg BW/day | Kumar et al. 2016a |
| Wistar rat, male | Copper sulfate | Feed | Daily up to 90 days | ND | 100 or 200 | Neurobehavior, enzymatic activity, total antioxidant capacity | Oxidative stress highest in the liver | LOAEL: 100 mg/kg BW/day | Kumar et al. 2016b |
| Sprague Dawley rat, male | Copper nanoparticle (327 nm), copper microparticle (253 µm) | Gavage | Daily for 2 weeks | ND | 0, 50, 100, 200, 400, 800 | General toxicity, biodistribution | Higher levels of Cu found in blood, organs, and toxicity to red blood cells, thymus, spleen, liver, kidney | NOAEL (Cu NP): 100 mg/kg/day; NOAEL (Cu MP): ≥400 mg/kg/day | Lee et al. 2016 |
| Rabbit sperm | Copper sulfate | *In vitro* | Sperm incubated for 48 hours in copper solution | 3.57, 3.63, 3.70, 3.77, 3.84, 4.85 | ND | Mobility | Decreased mobility, sperm death | LOAEL (mobility): 3.7 mg/L | Roychoudhury et al. 2010 |
| Zebrafish embryo | Copper sulfate | Aqueous exposure | 72-hour exposure to test solutions | 0.011, 0.014, 0.024, 0.068, 0.152, 0.364 | ND | Morphological effects | Lowest Cu concentration tested (0.01 mg/L) caused neuromast damage | EC_50_ post fertilization: 0.018 mg/L | Sonnack et al. 2015 |
| Wistar and SHR rat, male | Copper sulfate | Feed | Feed for 15 weeks | 100 | 8.2 | Raised blood pressure and hemoglobin levels | Elevated systolic blood pressure and hemoglobin levels | LOAEL: 8.2 mg/kg BW/day (only dose tested) | ECHA 2008 |
| **Human Studies** | | | | | | | | | |
| Adult women  (n = 60) | Copper sulfate | Water | 0, 1, 3, and 5 mg/L in tap water for two weeks followed by one week of tap water without copper | 0, 1, 3, 5 | ND | Gastrointestinal symptoms: abdominal pain, nausea, vomiting, diarrhea, copper homeostasis, liver function | Acute (GI) symptoms increased at >3 mg/L; no significant differences in effects between different copper ratios | NOAEL: 2 mg/L | Pizarro et al. 1999 |
| Adult women  (n = 45) | Copper sulfate: copper (II) oxide ratios | Water | 5 mg/L in tap water for one week followed by one week without copper in tap water, alternating for a total of 9 weeks, double-blind study | Ratios of soluble (copper sulfate) to insoluble (copper (II) oxide): 0:5, 1:4, 2:3, 3:2, 5:0 | ND | Liver function, gastrointestinal symptoms: abdominal pain, nausea, vomiting, diarrhea | No significant change to liver function, 4-fold increase in GI symptoms compared to “low-copper” tap water, 6/12 diarrhea episodes occurred during first week, enzymatic activity did not change between beginning and end, both copper compounds caused 54% nausea | ND | Pizarro et al. 2001 |
| Adult men and women  (n = 179) | Copper sulfate | Water | Weekly dose for five weeks | 0, 2, 4, 6, 8 | ND | Gastrointestinal symptoms: abdominal pain, nausea, vomiting, diarrhea | Nausea first and most common symptom reported; occurring within 15 min of ingestion | LOAEL: 6 mg/L; NOAEL 4 mg/L, both for GI effects and nausea | Araya et al. 2001 |
| Adult women  (n = 269) | Copper sulfate | Water | Single dose in bottled water | 0, 0.4, 0.8, 1.2, 1.6 mg Cu in 200 mL bottled spring water (0, 2, 4, 6 8) | ND | Gastrointestinal symptoms: abdominal pain, nausea, vomiting, diarrhea | Nausea first and most common symptom reported; occurring within 15 min of ingestion | LOAEL (nausea): 6 mg/L; NOAEL: 4 mg/L | Araya et al. 2003a |
| Adult men and women  (n = 1,365) | Copper sulfate | Water | Daily dose for two months, water prepared for drinking and food preparation | <0.01, 2, 4, 6 | ND | Gastrointestinal symptoms, homeostasis, liver function | Gastrointestinal effects increased at 6 mg/L, no effects for other endpoints | LOAEL (nausea): 6 mg/L | Araya et al. 2003b |
| Infants  (n = 148) | Copper sulfate | Formula made with water containing copper, or breast-fed from mothers ingesting copper in drinking water; after weaning, drinking water | Formula-fed or breast-fed between 3–12 months | <0.1 or 2 (water) | ND | Gastrointestinal symptoms, liver function, biochemical parameters, | No acute or chronic effects at 2 mg/L | No effect level: 2 | Olivares et al. 1998 |
| Adult men and women  (n = 61) | Copper sulfate | Water or juice | Weekly dose administered in water or orange-flavored juice for up to 12 exposures | 0, 2, 4, 6, 8, 10, 12 | ND | Gastrointestinal symptoms: abdominal pain, nausea, vomiting, diarrhea | Nausea and vomiting reported | In water NOAEL (nausea): 2 mg/L, NOAEL (vomiting): 4 mg/L; in orange flavored drink NOAEL (nausea): 8 mg/L, LOAEL (nausea): 4 mg/L | Olivares et al. 2001 |
| Adult men and women  (n = 7) | Copper gluconate | Pill | 1 pill/day for 12 weeks, double-blind study | 10 mg/day | ND | Liver function and gastrointestinal effects | Liver function was normal and no gastrointestinal effects quantified | NOAEL: 10 mg/day | Pratt et al. 1985 |
| Adult men  (n = 1) | Copper tablet, form not provided | Pill | Daily intake for 2 years of tablet, followed by higher dose for additional but unspecified time period | 30 mg/day for 2 years, 60 mg/day for additional time | ND | Liver function | Acute liver failure | LOAEL: 30 mg/day | ECHA 2008 |

^a^ Unless otherwise noted.

^b^ Only values for males are reported unless otherwise noted.

ALT is alanine amino-transferase.

BW is body weight.

NA is not applicable.

ND is not determined.

EC_50_ is the effective concentration inducing a response halfway between the baseline and maximum responses.

Where copper sulfate is CuSO_4_.

**Table S-3.** Summary of copper mutagenicity studies

| **Test Method** | **Cu Form** | **Dose** | **Model Organism** | **Test Method** | **Endpoint Measured** | **Results** | **Reference** |
| --- | --- | --- | --- | --- | --- | --- | --- |
| *in vitro* | Copper sulfate | 1 mg/plate | *Salmonella typhimurium* | Ames test | Mutagenicity; cytotoxicity | Non-mutagenic; highest concentrations caused cytotoxicity | EHA 2008 |
| *in vitro* | Copper sulfate | Up to 500 mg/L | Human fibroblasts and Chinese hamster ovary cells | 24-hour mammalian cell assay | Mutagenicity; cytotoxicity | DNA damage at high doses that also cause cytotoxicity (250–500 mg/L); low concentrations did not cause mutagenicity | EHA 2008 |
| *in vitro* | Copper sulfate | Up to 250 mg/L | Rat hepatocytes | 3-hour mammalian cell assay | Mutagenicity; cytotoxicity | DNA single strand breaks; however, no effects were observed for non-cytotoxic concentrations | EHA 2008 |
| *in vivo* | Copper sulfate | Selected 60% of LD_50_ at 745 mg Cu/kg/day; orally treated with 113.76 mg Cu/kg for two consecutive days | Five male and five female CD-1 mice: erythrocytes from sampled bone marrow | Micronucleus test | Mutagenicity; cytotoxicity | Did not cause an increase in micronucleus formation in polychromatic, though the dose did cause some toxicity | unpublished; in ECHA 2008 |
| *in vivo* | Copper sulfate | Oral with a single dose of 161 mg Cu/kg (632.5 mg/kg) or 509 mg Cu/kg (2,000 mg/kg) | Male Wistar rats | Unscheduled DNA synthesis (UDS) test; 2–4 hours or 12–14 hours | Mutagenicity; cytotoxicity | Did not cause DNA damage in hepatocytes under test conditions | unpublished; in ECHA 2008 |

**Table S-4.** Additional details for oral dose studies from Table S-2 that were selected for further consideration for the RfD calculation. Study endpoints focus on gastrointestinal and liver effects.

|  |  |  |  | **Dose** | |  |  |  |  |  |  |
| --- | --- | --- | --- | --- | --- | --- | --- | --- | --- | --- | --- |
| **Model Organism** | **Cu Form** | **Type of Testing** | **Treatment** | **Diet or Water Concentration (ppm)^a^** | **mg/kg BW/day^b^** | **Measurements** | **Endpoints** | **Results Summary** | **LOAEL or NOAEL** | **Reference** |  |
| **Animal Studies** | | | | | | | | | | | |
| Sprague Dawley rat, 30 male and 30 female in each dosing group | Copper sulfate pentahydrate | Reproduction toxicity: gonadal function, conception, parturition, growth, development, two-generation study with at least one set of litters produced/ generation | P1 male= 70 days prior to mating;  P1 females = 70 days prior to mating plus through pregnancy and until F1 weaning (21 days postpartum); F1 male = weaning through mating; F1 females = weaning through mating plus through pregnancy and until F2 weaning (21 days postpartum) | 0, 25.4, 127, 254, 381 mg/kg | Dependent on generation, Range: 1.53–55.7 (mg Cu/kg body weight/d) | Weekly: body weight and food consumption; Days 0, 4, 7, 14, 21: litters examined for number of live/dead pups, pup/litter weights, abnormal appearance or behavior, pathological exams, histopathological examination of tissue, sperm parameters | Spleen weight, reproductive toxicity | No effects on sperm, estrous cycle, mating, precoital interval, fertility, gestation length, implantation efficiency or site number, number of pups born, born alive, sex ratio, survival, organ weights, adverse effects at 381 mg/kg included decreased spleen weight | NOAEL (spleen growth): 15.2–26.7 mg/kg BW/day;  NOAEL (reproductive toxicity):  43.8 mg/kg BW/day (during premating)  26.5 mg/kg BW/day (during gestation)  55.7 mg/kg BW/day (during lactation) | ECHA 2008 |  |
| B6C3F_1_ mouse, male and female, 5 males and 5 females in each group | Copper sulfate | General toxicity | Drinking water for 15 days (5/sex/group) | 76, 254, 762, 2,543, 7,629 | 10, 24, 58, N/A, 367 for male; 15, 36, 62, N/A, 330 for female | Histopathological examination, mortality, water consumption | Reduced water consumption, body and organ weight, clinical toxicity symptoms (emaciation, weight loss, hypoactivity, dyspnea, tremors) related to dehydration, death | Increased mortality weight loss, and toxicity above 762 mg/L, all animals died at higher than 762 mg/L, effects due to decreased water intake from poor palatability | LOAEL (general toxicity): 762 mg/L | Hébert et al. 1993; Hébert 1993 |  |
| Fischer rat, male and female, 5 males and 5 females in each group | Copper sulfate | General toxicity | Drinking water for 15 days, *ad libitum* | 76, 254, 762, 2,543, 7,629 | 10, 29, 45, 36, 97 for male; 10, 26, 31, 31, 71 for female | Histopathological examination, mortality, water consumption | Reduced water consumption, body and organ weight, clinical toxicity symptoms (emaciation, hypoactivity, dyspnea, tremors) related to dehydration, death | Cu consumption ranged from 10–97 mg/kg BW/day, increased mortality and toxicity above 762 mg/L, effects due to decreased water intake from poor palatability, changes to kidneys in male rat at 10 and 29 mg/kg BW/day | LOAEL (general toxicity): 762 mg/L | Hébert et al. 1993; Hébert 1993 |  |
| B6C3F_1_ mouse, male and female, 5 males and 5 females in each group | Copper sulfate | General toxicity | Feed for 15 days | 255, 509, 1,018, 2,036, 4,072 | 42.8, 92.1, 196.8, 293.7, 717 for male; 53.5, 103.8, 216.1, 397.8, 780.8 for female | Histopathological examination, body weight, mortality, food consumption | Forestomach lesions | Forestomach lesions (2,036 and 4,076 mg/kg), liver, kidney, bone marrow, no significant change to body or organ weight | NOAEL (forestomach lesions): 42.8 and 53.5 mg/kg BW/day for male and female | Hébert et al. 1993; Hébert 1993 |  |
| Fischer rat, male and female, 5 males and 5 females in each group | Copper sulfate | General toxicity | Feed for 15 days | 255, 509, 1,018, 2,036, 4,072 | 23.4, 45.8, 92.4, 197.8, 324.5 for male; 22.7, 44.3, 93.4, 195.7, 285.3 for female | Histopathological examination, body weight, mortality, food consumption | Forestomach lesions, bone marrow/spleen depletion | Forestomach lesions (509 mg/kg), reduced body weight in males/females for two highest doses, decreased food consumption, bone marrow/spleen cell depletion at 2,036 and 4,076 mg/kg in males and females, no deaths occurred | NOAEL (forestomach lesions): 23.4 and 22.7 mg/kg BW/day for male and female | Hébert et al. 1993; Hébert 1993 |  |
| B6C3F_1_ mouse, male and female, 10 males and 10 females in each group | Copper sulfate | General toxicity | Feed for 13 weeks, *ad libitum* | 255, 509, 1,018, 2,036, 4,072 | 44, 97.2, 187.3, 397.8, 814.7 for male; 52.2, 125.7, 166.7, 536, 1,058 for female | Histopathological examination, body weight, mortality, food consumption, hematological examination, enzyme activity, urine analysis | Forestomach lesions | Forestomach lesions no liver or kidney effect, no effect on reproduction parameters | NOAEL: 97.2 and 125.7 mg/kg BW/day for male and female | Hébert et al. 1993; Hébert 1993 |  |
| Fischer rat, male and female, 10 males and 10 females in each group | Copper sulfate | General toxicity | Feed for 13 weeks, *ad libitum* | 127, 255, 509, 1,018, 2,036 | 8.1, 16.3, 32.8, 65.9, 140.2 for male; 8.7, 17.3, 34.4, 68.0, 134.4 for female | Histopathological examination, body weight, mortality, food consumption, hematological examination, enzyme activity, urine analysis | Forestomach lesions, liver toxicity, kidney toxicity | Changes to forestomach, liver (chronic inflammation) and kidney (toxicity) lesions, changes in urine chemistry, no effect on reproductive parameters, kidney changes more marked in males | NOAEL (forestomach): 16.3 and 17.3 mg/kg BW/day for male and female; NOAEL (liver and kidney): 16.3 mg/kg BW/day for male | Hébert et al. 1993; Hébert 1993 |  |
|  | | | | | | | | | | | |
| **Human Studies** | | | | | | | | | | | |
| Adult women, n = 60, Chile | Copper sulfate | Chronic effects | 0, 1, 3, 5 mg/L in tap water for two weeks followed by tap water without copper for one week, "Latin Square" design | 0, 1, 3, 5 | ND | Copper homeostasis (serum and ceruloplasmin), liver (serum and enzymes), GI symptoms | Gastrointestinal symptoms: abdominal pain, nausea, vomiting, diarrhea, copper homeostasis, liver function | Acute (GI) symptoms increased at >3 mg/L; no significant differences in effects between different copper ratios, response threshold between 1 and 3 mg/L | NOAEL: 2 mg/L | Pizarro et al. 1999 |  |
| Adult women, n = 45, Chile | Copper sulfate: copper (II) oxide ratios | Chronic effects | 5 mg/L in tap water for one week followed by tap water without copper for one week, alternating for a total of 9 weeks, double-blind and "Latin-square" design study | Soluble copper: 0.1, 0.9, 1.8, 2.7, 4.7  Total copper: 4.7, 5.3, 4.7, 5.2, 5.1 | ND | Self-reported GI effects, copper homeostasis (serum and ceruloplasmin), liver (serum and enzymes) | Gastrointestinal symptoms: abdominal pain, nausea, vomiting, diarrhea | Acute (GI) symptoms increased at ≥3 mg/L; no significant differences in effects between different soluble and insoluble copper ratios, 4-fold increase in GI symptoms compared to "low-copper" tap water, 6/12 diarrhea episodes occurred during first week, no change to liver function | ND | Pizarro et al. 2001 |  |
| Adult men and women, n = 179, USA, UK, Chile | Copper sulfate | Chronic effects | Weekly dose for five weeks in distilled, deionized water after an overnight fast, administered in 200 mL bolus | 0, 2, 4, 6, 8 | ND | Gastrointestinal effects recorded at 15 min, 1 hr, and 24 hr after ingestion | Gastrointestinal symptoms: abdominal pain, nausea, vomiting, diarrhea | Nausea first and most common symptom reported; occurring within 15 min of ingestion at following percentages 2% (0 mg/L), 1% (2 mg/L), 5% (4 mg/L)< 10% (6 mg/L), 18% (8 mg/L), abdominal pain at 4.5% (0 mg/L), 4% (2 mg/L), 8% (4 mg/L), 13% (6 mg/L), 25% (8 mg/L), symptoms less prevalent in men and in Chile | LOAEL: 6 mg/L; NOAEL: 4 mg/L, both for GI effects and nausea | Araya et al. 2001 |  |
| Adult women, n = 249, Chile, USA, Northern Ireland, China | Copper sulfate | Acute effects | Single bolus dose in bottled water | 0, 0.4, 0.8, 1.2, 1.6 mg Cu in 200 mL bottled spring water (0, 2, 4, 6, and 8) | ND | Gastrointestinal effects recorded at 15 min, 1 hr, and 24 hr after ingestion | Gastrointestinal symptoms: abdominal pain, nausea, vomiting, diarrhea | Nausea first and most common symptom reported; occurring within 15 min of ingestion | LOAEL (nausea): 6 mg/L; NOAEL: 4 mg/L | Araya et al. 2003a |  |
| Adult men and women, n = 1,365 | Copper sulfate | Chronic effects | Daily dose for two months, water prepared and used for drinking and food preparation | <0.01, 2, 4, and 6 | ND | Self-reported GI effects | Gastrointestinal symptoms, homeostasis, liver function | Gastrointestinal effects increased at 6 mg/L, no effects for other endpoints | LOAEL (nausea): 6 mg/L | Araya et al. 2003b |  |
| Infants | Copper sulfate | Chronic and acute effects | Received drinking water with copper between 3–12 months, formula containing drinking water with copper, or breast-fed from mothers ingesting copper in drinking water, after weaning, ingestion through drinking water | <0.1 or 2 | ND | Serum level, ceruloplasmin level, erythrocyte enzyme activity, metallothionein; liver function via enzymatic activity, serum bilirubin; gastrointestinal symptoms, and respiratory disorder | Gastrointestinal symptoms, liver function, biochemical parameters | No acute or chronic effects at 2 mg/L | NOAEL: 2 mg/L | Olivares et al. 1998 |  |
| Adult men and women (men n = 47, women n = 61), Chile | Copper sulfate | Acute effects | Weekly dose administered in 200 mL water or orange-flavored juice for up to 12 exposures | 0, 2, 4, 6, 8, 10, 12 | ND | Water consumption recorded for mothers, gastrointestinal effects, respiratory disorder (infants only), blood samples measured for: serum level, ceruloplasmin level, erythrocyte enzymatic activity, metallothionein; liver function tests: enzymatic activity, serum bilirubin | Gastrointestinal symptoms: abdominal pain, nausea, vomiting, diarrhea | Nausea confirmed in 76% volunteers and more frequent at higher doses, nausea and vomiting reported, nausea percentages at specific doses were 9% (4 mg/L), 10% (6 mg/L), 19% (8 mg/L), 19% (10 mg/L), 21% (12 mg/L), nausea plus vomiting occurred at less frequency than nausea alone, only one case of diarrhea and no cases of abdominal pain | In water NOAEL (nausea): 2 mg/L, LOAEL (nausea): 4 mg/L; NOAEL (vomiting): 4 mg/L; in orange flavored drink NOAEL (nausea): 6 mg/L, NOAEL (vomiting): 12 mg/L | Olivares et al. 2001 |  |

^a^ Unless otherwise noted.

^b^ Only values for males are reported unless otherwise noted.

BW is body weight.

NA is not applicable.

ND is not determined.

Where copper sulfate is CuSO_4_.

# References

(see main article for references cited in both main text and SI; references below are specific to the SI)

Aburto, E.M., A.E. Cribb, I.C. Fuentealba, B.O. Ikede, F.S. Kibenge, and F. Markham. 2001. Morphological and biochemical assessment of the liver response to excess dietary copper in Fischer 344 rats. Can. J. Vet. Res. 65(2):97.

Aoyagi, S., and D.H. Baker. 1993. Nutritional evaluation of a copper-methionine complex for chicks. Poult. Sci. 72(12):2309–2315.

Arnal, N., L. Dominici, M.J. de Tacconi, and C.A. Marra. 2014. Copper-induced alterations in rat brain depends on route of overload and basal copper levels. Nutrition 30(1):96–106.

Chung, M.K., S.S. Baek, S.H. Lee, H. Kim, K. Choi, and J.C. Kim. 2009. Combined repeated dose and reproductive/developmental toxicities of copper monochloride in rats. Environ. Toxicol. 24(4):315–326.

EBRC. 2007. Health risk assessment guidance for metals (HERAG): gastrointestinal uptake and absorption, and catalogue of toxicokinetic models. Fact Sheet. August.

Haywood, S., and M. Loughran. 1985. Copper toxicosis and tolerance in the rat. II. Tolerance–a liver protective adaptation. Liver Int. 5(5):267–275.

Hristozov, D., L. Pizzol, G. Basei, A. Zabeo, A. Mackevica, S.F. Hansen, I. Gosens, F.R. Cassee, W. de Jong, A.J. Koivisto, N. Neubauer, A.S. Jimenez, E. Semenzin, V. Subramanian, W. Fransman, K.A. Jensen, W. Wohlleben, V. Stone, and A. Marcomini. 2018. Quantitative human health risk assessment along the lifecycle of nano-scale copper-based wood preservatives. Nanotoxicol 12(7): 747–765.

Klimisch, H.J., M. Andreae, and U. Tillmann. 1997. A systematic approach for evaluating the quality of experimental toxicological and ecotoxicological data. Regul. Toxicol. Pharmacol. 25(1):1–5.

Kumar, V., J. Kalita, U.K. Misra, and H.K. Bora. 2015. A study of dose response and organ susceptibility of copper toxicity in a rat model. J. Trace Elem. Med. Biol. 29:269–274.

Kumar, V., J. Kalita, H.K. Bora, and U.K. Misra. 2016a. Temporal kinetics of organ damage in copper toxicity: A histopathological correlation in rat model. Regul. Toxicol. Pharmacol. 81:372–380.

Kumar, V., J. Kalita, H.K. Bora, and U.K. Misra. 2016b. Relationship of antioxidant and oxidative stress markers in different organs following copper toxicity in a rat model. Toxicol. Appl. Pharmacol. 293:37–43.

Ledoux, D.R., P.R. Henry, C.B. Ammerman, P.V. Rao, and R.D. Miles. 1991. Estimation of the relative bioavailability of inorganic copper sources for chicks using tissue uptake of copper. J. Anim. Sci. 69(1):215–222.

Ledoux, D.R., E.B. Pott, P.R. Henry, C.B. Ammerman, A.M. Merritt, and J.B. Madison. 1995. Estimation of the relative bioavailability of inorganic copper sources for sheep. Nutr. Res. 15(12):1803–1813.

Lee, I.C., J.W. Ko, S.H. Park, N.R. Shin, I.S. Shin, C. Moon, J.H. Kim, H.C. Kim, and J.C. Kim. 2016. Comparative toxicity and biodistribution assessments in rats following subchronic oral exposure to copper nanoparticles and microparticles. Part. Fibre Toxicol. 13(1):56.

Lönnerdal, B., J.G. Bell, and C.L. Keen. 1985. Copper absorption from human milk, cow’s milk, and infant formulas using a suckling rat model. Am. J. Clin. Nutr. 42(5):836–844.

Miles, R.D., S.F. O’keefe, P.R. Henry, C.B. Ammerman, and X.G. Luo. 1998. The effect of dietary supplementation with copper sulfate or tribasic copper chloride on broiler performance, relative copper bioavailability, and dietary prooxidant activity. Poult. Sci. 77(3):416–425.

Platten III, W.E., T..P. Luxton, T. Gerke, S. Harmon, N. Sylvest, K. Bradham, and K. Rogers. 2014. Release of microionized copper particles from pressure-treated wood products. EPA report NMo. EPA/600/R-14/365. September.

Roychoudhury, S., P. Massanyi, J. Bulla, M.D. Choudhury, L. Straka, N. Lukac, G. Formicki, M. Dankova, and L. Bardos. 2010. *In vitro* copper toxicity on rabbit spermatozoa motility, morphology and cell membrane integrity. J. Environ. Sci. Health, Part A. 45(12):1482–1491.

Sonnack, L., S. Kampe, E. Muth-Köhne, L. Erdinger, N. Henny, H. Hollert, C. Schäfers, and M. Fenske, M. (2015). Effects of metal exposure on motor neuron development, neuromasts and the escape response of zebrafish embryos. Neurotoxicol. Teratol. 50:33–42.

Vasconcelos, T., B. Sarmento, and P. Costa. 2007. Solid dispersions as strategy to improve oral bioavailability of poor water soluble drugs. Drug Discovery Today 12(23):1068–1075.
